# Kv12.1 (*Kcnh8*) maintains hair cell potassium homeostasis and protects against noise-induced hearing loss in mice

**DOI:** 10.1172/JCI202988

**Published:** 2026-11-16

**Authors:** Yaoqian Liu, Yanyan Jia, Jiaoyao Ma, Xian Gao, Yunjie Li, Yu Dong, Mingxuan Wu, Gaogan Jia, Yikang Huang, Yiyun Lou, Mengrou Xu, Tianshi Sun, Yuqiang Ding, Huawei Li, Mingyu Xia, Wenyan Li

**Affiliations:** 1 ENT institute and Otorhinolaryngology Department of Eye & ENT Hospital, State Key Laboratory of Medical Neurobiology and MOE Frontiers Center for Brain Science;; 2 Institutes of Biomedical Sciences;; 3 NHC Key Laboratory of Hearing Medicine; and; 4 The Institutes of Brain Science and the Collaborative Innovation Center for Brain Science, Fudan University, Shanghai, China.; 5 Shanghai Key Laboratory of Gene Editing and Cell Therapy for Rare Diseases, Shanghai, China.; 6 School of Life Sciences, Zhejiang Chinese Medical University, Hangzhou, China.; 7 Laboratory Animal Center, Fudan University, Shanghai, China.; 8 Shanghai Engineering Research Centre of Cochlear Implant, Shanghai, China.

**Keywords:** Neuroscience, Otology, Potassium channels, Synapses

## Abstract

Potassium (K^+^) channels play fundamental roles in stabilizing resting membrane potential and regulating neuronal excitability. In sensory hair cells (HCs), a site where mechanoelectrical transduction requires exquisite electrochemical stability, the maintenance of K^+^ equilibrium is particularly critical. Nevertheless, the key channels that preserve this homeostasis under pathological stress remain poorly defined. Through single-nucleus RNA sequencing of the adult mouse cochlea, we identified *Kcnh8*, which encodes the voltage-gated potassium channel Kv12.1, as the predominant KCNH family member exclusively and highly expressed in HCs. Its expression pattern is evolutionarily conserved and abundant in human fetal cochlea. Loss of *Kcnh8* in mice causes progressive hearing loss, featuring disorganized hair bundles and synaptic defects. *Kcnh8* deficiency caused intracellular K^+^ accumulation, leading to HC hyperexcitation. This, in turn, triggered aberrant calcium influx and attenuated synaptic vesicle release, highlighting the critical role of Kv12.1 in maintaining K^+^ homeostasis. Pathologically, *Kcnh8*-deficient mice exhibited increased susceptibility to noise exposure, which exacerbated HC damage and hearing impairment. Importantly, AAV-mediated delivery of *Kcnh8* gene rescued noise-induced hearing loss, mitigated intracellular K^+^ accumulation, and protected HC synapses. Our findings establish Kv12.1 as a central regulator of HC K^+^ homeostasis and highlight its therapeutic potential for preventing noise-induced hearing loss.

## Abstract 2

Potassium channel Kv12.1 protects inner-ear hair cells. Without it, hearing declines; gene therapy restores potassium levels and prevents noise-induced hearing loss.

## Graphical abstract

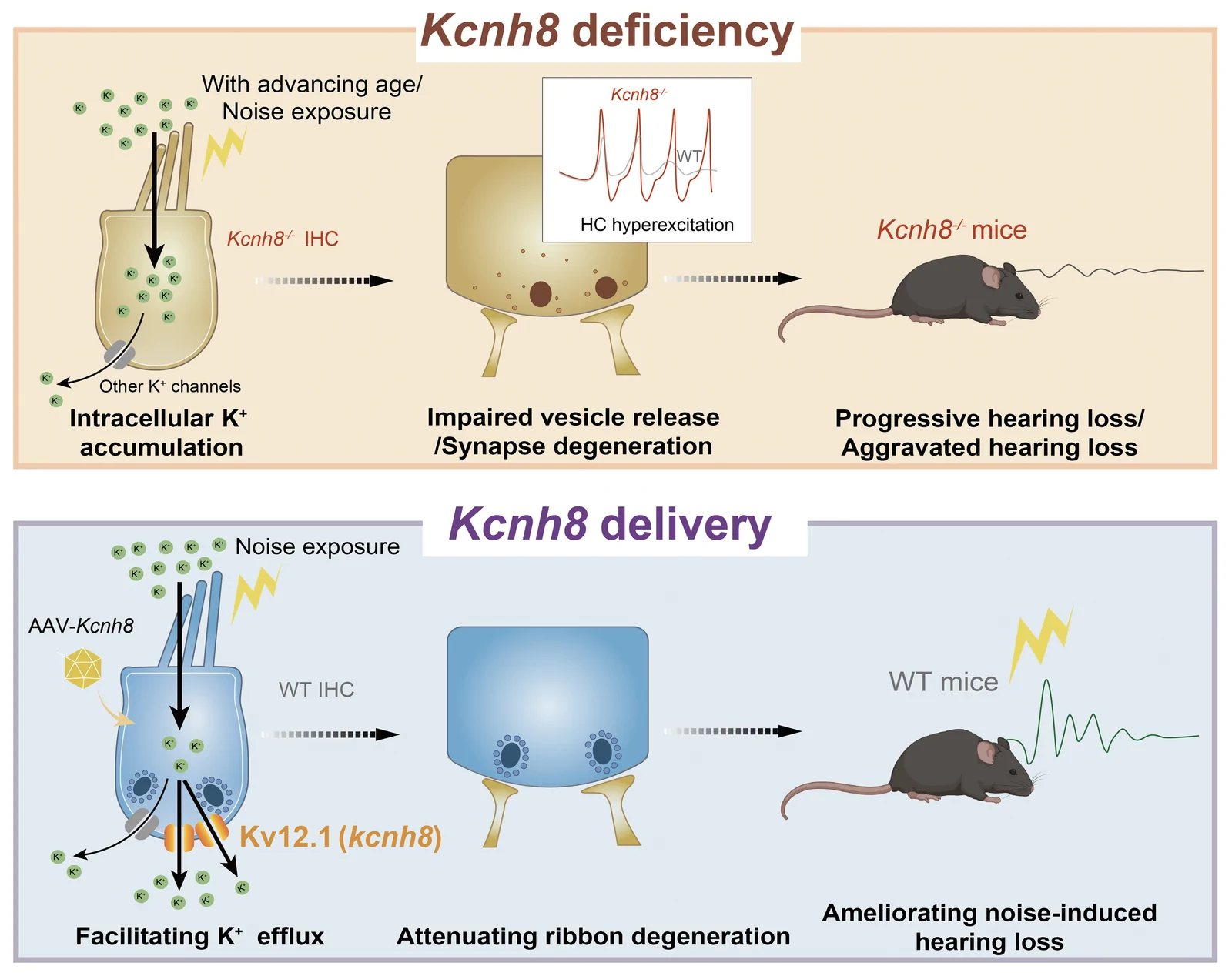

## Introduction

As a pressing global health burden, sensorineural hearing loss predominantly stems from cochlear mechanosensory hair cell (HC) damage induced by factors like noise exposure, aging, genetic mutations, and ototoxic drugs (1, 2). The distinct morphological and physiological characteristics of HCs underlie our ability to perceive sound and navigate the surrounding environment (3). Ion channels in HCs are pivotal for normal acoustic-electric signals’ conversion while driving pathological HC degeneration (4–6). As the phylogenetically most diversified ion channel superfamily, potassium (K^+^) channels segregate into families/subfamilies defined by sequence homology, with voltage-gated K^+^ (Kv) channels constituting the largest and most diverse subfamily among the ion channels (7). Especially, KCNH voltage-dependent K^+^ channels critically sustain cellular excitability and propel multisystem pathogenesis (8–10), while their specific roles in cochlear HC and hearing function persist as a fundamental knowledge gap.

The KCNH ion channel family — phylogenetically conserved from nematodes to humans — comprises 3 structurally distinct subfamilies: ether-à-go-go (EAG), EAG-related gene (ERG), and EAG-like K^+^ (ELK) channels (11, 12). Leveraging a single-nucleus RNA-sequencing (snRNA-seq) atlas of adult mouse cochlea generated by our team (13), we systematically profiled Kv channel–encoded genes. *Kcnh8* (encoding Kv12.1) exhibits unique enrichment in cochlear sensory HCs among all KCNH channels, with near-exclusive expression in inner hair cells (IHCs) and outer hair cells (OHCs) compared with other cell types of the organ of Corti. Kv12.1 is classified within the ELK subfamily, yet the physiological role and native functional correlates of ELK channels remain poorly defined compared with ERG or EAG channels. As an ELK subfamily member, Kv12.2 channel (encoded by *Kcnh3*) exhibits a fast inactivation component current (14), and its deficiency drives persistent neuronal hyperexcitability manifested by frequent interictal spiking and spontaneous seizures (9). Diverging from Kv12.2’s fast inactivation, Kv12.1 mediates slowly activating, noninactivating potassium currents, consequently generating a sustained outward conductance at resting or near-threshold potentials (15). Although the biophysical properties of the Kv12.1 channel have been characterized, its functional significance in physiology and disease is largely unexplored. To date, only one investigation has demonstrated that the Kv12.1 channel in suprachiasmatic nucleus neurons modulates mean repetitive firing rates of nighttime, suggesting a conserved function in day/night rhythm regulation (16). However, the physiological function of Kv12.1 in any system, including the auditory pathway, is largely unexplored.

During hearing initiation, cochlear HCs convert sound waveforms into receptor potentials through mechanoelectrical transduction (17). In auditory physiology, the resting potential and excitability of HCs are shaped by several K^+^ currents. In HCs, the large-conductance, voltage- and Ca^2+^-activated K^+^ (BK) channel current dominates the fast repolarization phase (18), while the Kv7.4 channel (encoded by *Kcnq4*) underlies a negative-activating current that stabilizes the OHC resting potential (19–21). As the leading etiological factor in HC damage, noise trauma drives cochlear pathogenesis critically linked to dysregulation of K^+^ homeostasis (22, 23). Targeted modulation of BK and Kv7.4 channels holds therapeutic promise for noise-induced hearing loss (24, 25). The absence of clinically viable targeted therapies represents an unmet medical need. Although Kv12.1 has been proposed to mediate the slow delayed rectifier K^+^ current and shows relatively HC-specific expression, its physiological significance remains undefined (26). Given its biophysical profile and specific expression, its role in auditory function and noise-induced hearing loss warrants systematic investigation.

Here, we demonstrate that *Kcnh8* is essential for maintaining K^+^ homeostasis in HCs. Its loss leads to progressive hearing loss, synaptic deficits, and increased susceptibility to noise-induced damage. Crucially, targeted *Kcnh8* gene delivery protected against noise-induced hearing loss, nominating Kv12.1 as a compelling therapeutic target.

## Results

### The evolutionarily conserved and exclusive expression of Kcnh8 in cochlear HCs.

To determine the cochlear expression pattern of KCNH family genes, we analyzed inner ear snRNA-seq data from adult mice (13). Among the 50 potassium channel genes that were differentially expressed across all cochlear cell types, *Kcnh8* exhibited the highest and most specific expression in hair cells, and it was preferentially expressed in IHCs and OHCs (Figure 1, A and B). *Kcnh8* encodes the Kv12.1 potassium channel, which belongs to the ELK subfamily of the KCNH channel family (Figure 1C). To comprehensively determine the spatiotemporal expression pattern of *Kcnh8* in the cochlea, we performed RNAscope in situ hybridization on cochlear sections from wild-type (WT) mice at embryonic days (E) 13.5 and 18.5, as well as postnatal days (P) 1 and 14, and 1 month (M) (Figure 1, D and E). At E13.5, *Kcnh8* expression was diffusely distributed throughout the entire cochlear duct (Figure 1E). From E18.5 to P14, the signals in HCs, especially in IHCs, increased in expression and gradually intensified, but signals were also detected in cells surrounding the HCs, reaching relative exclusivity by 1 M (Figure 1, E and F). Furthermore, analysis of both cochlear sections and whole-mount preparations further confirmed the abundant and specific expression of *Kcnh8* in HCs, which was consistent along the base-to-apex turns of the 1 M cochlea (Figure 1D and Supplemental Figure 1, A and B; supplemental material available online with this article; https://doi.org/10.1172/JCI202988DS1). This nonspecific HC expression pattern of *Kcnh8* during development was further confirmed by single-cell RNA-seq (scRNA-seq) analysis from P1–P7 mouse data (27) (Supplemental Figure 1C). Together, these data reveal a dynamic yet specific expression profile for *Kcnh8*, which is the predominant KCNH family member in cochlear HCs and whose localization is scattered throughout development, culminating in a stable pattern in adulthood.

To investigate the evolutionary conservation of *Kcnh8*, we compared its amino acid sequence across several representative species used in auditory research. The sequences were found to be highly conserved (Figure 1G). Specifically, *Macaca mulatta* shared 99.19% sequence identity with humans, while mice and rats shared 91.92% and 91.73% identity with humans, respectively. Using RNAscope on cochlear sections from donated human fetuses, we detected a faint and scattered *KCNH8* mRNA signal within the SOX2-positive prosensory domain at 11 postconception weeks (PCW), prior to HC formation. By 17 PCW, *KCNH8* was robustly expressed in the differentiated IHCs and OHCs of the sensory domain, with additional expression in surrounding SOX2-positive supporting cells (Figure 1H). This spatiotemporal pattern in human fetuses recapitulates the expression pattern observed during HC development in mice. To determine the subcellular localization of the endogenous Kv12.1 protein, we employed an adeno-associated virus–mediated (AAV-mediated) homology-independent universal genome engineering (HiUGE) knock-in system (28). To tag the endogenous Kv12.1 protein encoded by *Kcnh8* with an HA-epitope, we employed a CRISPR/Cas9-mediated knock-in strategy using a dual-orientation payload design (Supplemental Figure 1D). To achieve this, cultured cochlear explants from Cas9-expressing mice were infected with the dual-AAV system (Figure 1I). Whereas no HA signal was detected in the absence of the system, transduced explants exhibited a robust HA signal in some HCs, confirming successful insertion (Figure 1J and Supplemental Figure 1E). The HA signal was localized to both the HC plasma membrane and the cytoplasm, indicating that the Kv12.1 protein is present in these cellular compartments (Figure 1J). Collectively, our study unveils *Kcnh8* as a highly specific potassium channel in cochlear hair cells and delineates its evolutionarily conserved spatiotemporal expression profile.

### Kcnh8 deficiency leads to progressive hearing loss.

To investigate the biological function of *Kcnh8* in the auditory system, we knocked out the gene by deleting its exon 4 using CRISPR/Cas9 technology (Figure 2A). *Kcnh8*-deficient mice were viable with normal survival across genotypes (data not shown). We confirmed decreased *Kcnh8* mRNA expression in homozygous and heterozygous knockout (KO) mice by qRT-PCR (Figure 2B). We further verified this knockout effect at the protein level, where we observed a genotype-dependent dosage effect of *Kcnh8*-encoded Kv12.1 expression (Figure 2C).

Next, we assessed the auditory function of *Kcnh8*^–/–^ and *Kcnh8^+/–^* mice using auditory brain stem response (ABR) measurements, as well as distortion product otoacoustic emissions (DPOAE) tests. As shown in Figure 2D, hearing loss was observed in *Kcnh8*-deficient mice at 1 M of age, evidenced by significantly elevated ABR thresholds in response to 24 and 32 kHz tone bursts (Figure 2D). The thresholds in both *Kcnh8*^–/–^ and *Kcnh8^+/–^* groups were approximately 10–20 dB higher than those in WT controls. This deficit worsened by 3 M, with *Kcnh8*^–/–^ mice showing the most pronounced threshold shifts while *Kcnh8^+/–^* mice exhibited an intermediate phenotype, indicating a genotype-dependent dosage effect (Figure 2D). Although ABR thresholds in response to clicks showed no significant difference at 1 M and 3 M, a significant hearing impairment emerged in *Kcnh8*-deficient mice by 6 M (Supplemental Figure 2A). Across specific frequencies (4–24 kHz), ABR thresholds were further elevated in both *Kcnh8*^–/–^ and *Kcnh8*^+/–^ mice, with statistically significant differences observed between the *Kcnh8*^–/–^ and WT groups (Figure 2D). We further analyzed the features of wave I of the ABR evoked by a 16 kHz tone at 90 dB SPL, which revealed that both *Kcnh8*^–/–^ and *Kcnh8*^+/–^ mice exhibited reduced amplitude and increased latency compared with controls (Figure 2, E and F). At 1 M, no DPOAE abnormality was detected in *Kcnh8*-deficient mice, suggesting an initial selective impairment of IHC function. However, progressively worsening DPOAE response, indicative of OHC dysfunction, emerged at 3 M and 6 M of age (Figure 2G and Supplemental Figure 2B).

Given the observed hearing deficits, we next assessed whether there were corresponding pathological changes in the cochlea of *Kcnh8*-deficient mice. Staining with the HC marker MYO7A revealed no significant difference in HC counts at 1 M. However, progressive OHC loss was observed in *Kcnh8*^–/–^ mice over time. Severe loss was first evident in the basal turn, where only a small fraction of OHCs remained by 6 M (Figure 2, H and I). The middle turn also showed significantly greater OHC loss in *Kcnh8*^–/–^ mice compared with WT at 6 M (Supplemental Figure 2, E and F). In contrast, no significant difference in HC counts was detected in the apical turn, nor were IHCs lost, in *Kcnh8*-deficient mice at any age (Figure 2, H and I, and Supplemental Figure 2, C–F). Collectively, these results demonstrate that *Kcnh8* plays an essential role in maintaining auditory function, and its deficiency underlies a progressive hearing loss.

### Kcnh8^–/–^ mice exhibit stereocilia loss and ribbon synapse degeneration in HCs.

To further characterize the ultrastructure in HCs affected by *Kcnh8* deficiency, we examined HC stereocilia and synaptic morphology. Scanning electron microscopy (SEM) revealed pronounced loss of OHC stereocilia in the basal turn of *Kcnh8*^–/–^ mice at 3 M and 6 M (Figure 3A). A milder stereocilia loss was also observed in the middle turn with *Kcnh8* deficiency at 6 M (Supplemental Figure 3A). We next characterized the stereocilia morphology on the remaining HCs. Unlike the orderly 3 rows of stereocilia seen in WT OHCs, we observed stereocilia loss in the basal turn of WT mice at 3 and 6 months of age (Figure 3A). This loss predominantly targeted the shortest row, a phenotype that was markedly exacerbated in the basal turn OHCs of *Kcnh8*^–/–^ mice at 3 M and 6 M (Figure 3, A and B). Similarly, significant stereocilia loss was observed in the middle turn of *Kcnh8*^–/–^ mice at these time points. In contrast, no obvious stereocilia loss was observed in IHCs of *Kcnh8*^–/–^ mice compared with controls (Figure 3, A and B, and Supplemental Figure 3, A and B). Our findings implicate the role of *Kcnh8* in maintaining stereocilia integrity, as its loss causes progressive degeneration, with OHCs being particularly vulnerable.

We next assessed the IHC ribbon synapses in the cochlea by immunostaining, using CtBP2 to label the presynaptic ribbons and HOMER1 for the postsynaptic densities. Quantification of synaptic puncta revealed no significant difference in the number of synaptic pairs between *Kcnh8*^–/–^ and WT mice across all cochlear turns at 1 M of age (Figure 3, C and D, and Supplemental Figure 3C). At 3 M, the number of synaptic pairs was significantly reduced in the basal turn of *Kcnh8*-deficient mice (Figure 3D). With advancing age, this synaptic loss progressed, and by 6 M, CtBP2/HOMER1 pairs were markedly decreased throughout the entire cochlea, from the base to the apex, compared with WT mice. We further used transmission electron microscopy (TEM) to examine the morphology of the ribbon synapses. In the basal turn of IHCs in 1 M WT mice, the synaptic vesicles (SVs) exhibited the characteristic garland-like arrangement around the synaptic ribbons (Figure 3E). Abnormal SV organization was already evident at 1 M, with SVs appearing disordered and irregularly dispersed around the ribbons in basal IHCs of *Kcnh8*^–/–^ mice. We quantified the number of SVs per synaptic ribbon and the distance of SVs from the ribbons. This analysis revealed that at 3 and 6 months, *Kcnh8*^–/–^ mice exhibited not only disorganized SVs but also a reduced SV count and an increased SV-to-ribbon distance compared with WT mice (Figure 3, E and F). This pathological pattern underscores the critical importance of *Kcnh8* in maintaining synaptic structural stability (Figure 3G).

### Kcnh8 disruption elevates early IHC activity and compromises mature synaptic function.

HC activation follows a sequence of events: K^+^ influx depolarizes the membrane to initiate vesicle release, a process balanced by K^+^ efflux that repolarizes the cell to maintain resting potential (Figure 4A). Kv12.1 has been proposed to contribute to the outward K^+^ conductance that regulates IHC receptor potentials (26). To directly evaluate its function, we performed whole-cell patch-clamp recordings on IHCs acutely isolated from P10 mouse cochleae (Supplemental Figure 4A). Cells were held at –90 mV and depolarized in 10 mV steps to +40 mV, followed by repolarization to –110 mV (Figure 4B). The results show that *Kcnh8*-KO IHCs displayed markedly reduced outward K^+^ currents compared with WT cells across –30 to +50 mV (Figure 4, B and C), indicating that *Kcnh8* deficiency substantially impairs outward K^+^ efflux during depolarization. Analysis of activation gating further showed a rightward shift of the conductance-voltage relationship in *Kcnh8*-KO IHCs (Figure 4D). These results indicate that *Kcnh8* initiates outward K^+^ efflux at the onset of IHC depolarization and becomes progressively more prominent as depolarization proceeds. To verify the bidirectional influence of *Kcnh8* in cochlear HCs, we overexpressed (OE) *Kcnh8* in HCs of P3 WT mice via AAVanc80L65 delivery and assessed the outcome at P10. As expected, the *Kcnh8*-OE IHCs exhibited significantly enhanced outward K^+^ currents compared with WT controls (Figure 4, E and F). Furthermore, the activation curve displayed a pronounced leftward shift (Figure 4G), and kinetic fitting revealed a reduced *V_half_* together with an increased slope factor (Figure 4H). These properties indicate that elevated *Kcnh8* expression promotes earlier and steeper activation, consistent with its role as a major outward rectifier during early depolarization.

As established in developing IHCs, outward K^+^ currents are the primary determinants of receptor potential amplitude and play a central role in regulating voltage-gated Ca^2+^ channel activation and action potential firing (29). We next recorded action potentials evoked by depolarizing current injections. *Kcnh8* deficiency IHCs generated significantly more action potentials than WT cells at equivalent current steps and exhibited a more depolarized resting membrane potential (Figure 4, I and J), reflecting heightened excitability. Single-spike analysis revealed faster depolarization rates and larger spike amplitudes in *Kcnh8* deficiency IHCs (Figure 4K and Supplemental Figure 4F), demonstrating that *Kcnh8* normally restrains IHC firing dynamics during pre–hearing development. Elevated action potential firing is expected to enhance voltage-gated Ca^2+^ entry and synaptic release. Indeed, Ca^2+^ currents were significantly larger in *Kcnh8*-KO P10 IHCs than in WT controls (Supplemental Figure 4, B and C), and sustained depolarizations evoked markedly greater membrane capacitance increments, indicating increased vesicle exocytosis (Supplemental Figure 4, D and E). Together, these findings indicate that impaired outward K^+^ efflux at P10 causes IHC hyperexcitability, thereby increasing Ca^2+^ influx and promoting excessive transmitter release during the critical period of synapse maturation.

Given that prolonged or excessive glutamate release can induce excitotoxic damage to IHC ribbon synapses and downstream auditory neurons (30, 31), we asked whether the early increase in excitability and transmitter release seen at P10 in *Kcnh8*-KO mice results in long-lasting synaptic dysfunction. We therefore evaluated Ca^2+^ currents and exocytic capacitance changes in IHCs from 1-month-old mice. In contrast with P10, *Kcnh8*-KO adult IHCs exhibited significantly reduced Ca^2+^ currents and smaller ΔCm responses compared with WT cells (Figure 4, L–O). We further examined K^+^ currents, evoked action potentials, Ca^2+^ currents, and vesicle release in heterozygous *Kcnh8*-deficient mice and found that, compared to WT mice, the results were consistent with those observed in homozygous mice (Supplemental Figure 5, A–H). Reduced Ca^2+^ currents and smaller ΔCm responses were also found in heterozygous *Kcnh8*-deficient HCs compared with WT HCs, indicating a dose-dependent effect of *Kcnh8* deficiency (Supplemental Figure 5, I–L). This functional decline suggests that early-life hyperexcitability and excessive synaptic vesicle release may impose lasting excitotoxic stress, ultimately impairing synaptic function in the mature cochlea.

### Kcnh8 deficiency increases susceptibility to noise-induced HC damage.

Given that hereditary deafness data do not implicate this gene and the phenotype is progressive, we sought to determine whether environmental stressors exacerbate the hearing loss caused by *Kcnh8* deficiency. WT and *Kcnh8*^–/–^ mice at 1 M were exposed to a moderate noise paradigm of 97 dB SPL for 2 hours to an 8–16 kHz narrow-band noise to induce threshold shifts (Figure 5A). ABR testing performed 1 day postexposure revealed a marked elevation in thresholds across 8–32 kHz in both genotypes compared with preexposure baselines, confirming successful induction of temporary threshold shift (Figure 5B). Notably, at 16 and 24 kHz, the threshold shifts in *Kcnh8*^–/–^ mice were significantly greater than those in WT littermates, by 14.37 ± 4.76 dB and 24.5 ± 4.04 dB SPL, respectively. As recovery progressed, a significant intergroup difference emerged at 32 kHz by day 3, with *Kcnh8*^–/–^ mice exhibiting thresholds 13.12 ± 3.65 dB SPL higher than WT controls. These differences between genotypes persisted from 16 to 32 kHz through day 14 (Figure 5B). Although WT mice showed substantial recovery across the 8–32 kHz range over time, the *Kcnh8*^–/–^ mice exhibited pronouncedly poorer recovery. Together, these results indicate that *Kcnh8* deficiency increases susceptibility to noise-induced hearing loss and impairs functional restoration after acoustic trauma.

We next examined the morphological differences in synaptic structure after noise exposure. On postnoise day 1, the number of synaptic pairs in the middle turn of IHCs was significantly reduced in both groups compared with their preexposure baselines (Figure 5, C and D). Furthermore, throughout the 14-day recovery period, *Kcnh8*^–/–^ mice consistently exhibited fewer CtBP2/HOMER1-labeled synaptic pairs in IHCs compared with WT mice at all time points checked, despite a gradual recovery trend in both genotypes. Ultrastructural analysis by TEM further identified exacerbated ribbon synapse degeneration in noise-exposed *Kcnh8*^–/–^ mice (Figure 5E). Specifically, from day 1 to day 14 postexposure, the synapses showed improperly aligned vesicles and a lower vesicle count around ribbons compared with WT (Figure 5, E and F). Corroborating this observation, further analysis of the vesicle reserve showed a significant decrease in the number of free vesicles in *Kcnh8*^–/–^ mice relative to WT controls (Figure 5F).

To determine if the noise vulnerability of *Kcnh8*^–/–^ HCs results from defective K^+^ efflux, we loaded dissected cochlear tissues with the K^+^ indicator EPG-2AM. On day 1 postexposure, both genotypes exhibited marked K^+^ accumulation in IHCs, with a nonsignificant increasing trend in *Kcnh8*^–/–^ versus WT (Figure 5, G and H). By day 14, however, WT IHCs had recovered to baseline K^+^ levels, while *Kcnh8*^–/–^ IHCs maintained a significantly higher K^+^ load. This persistent dysregulation demonstrates that *Kcnh8* deficiency causes hearing damage by disrupting hair cell K^+^ homeostasis. Compared with WT mice, *Kcnh8*^–/–^ mice had higher K^+^ concentration on day 7 and 14 after noise (Figure 5H). These results indicate that the auditory deficits in *Kcnh8*^–/–^ mice are due to a failure to restore K^+^ homeostasis in HCs.

### AAV-mediated delivery of Kcnh8 protects against noise-induced HC injury and preserves hearing function in WT mice.

Having established that Kv12.1 is essential for K^+^ efflux in hair cells, we next explored its therapeutic potential by targeting this channel to treat noise-induced hearing loss (Figure 6A). Given the delayed onset of AAV-mediated transgene expression in vivo, we verified the presence of exogenous *Kcnh8*-encoding protein in HCs to define the appropriate time point for subsequent noise exposure. Detection of the FLAG fusion tag indicated that robust exogenous protein expression appeared by day 7 postinjection and remained stable up to day 7 in *Kcnh8* delivery WT mice (Figure 6B). Furthermore, FLAG signal was detected in HCs across the apex, middle, and base cochlear turns on day 7 postinjection (Figure 6C). The absence of fluorescence in contralateral ears, together with no hearing loss on day 7 postinjection, confirms the targeted expression and safety of the AAV delivery procedure (Supplemental Figure 6, A and B). We confirmed successful *Kcnh8* overexpression in HCs by RT-PCR, Western blot, and RNAscope, which all showed elevated *Kcnh8* mRNA and protein levels in the delivery ears relative to the contralateral ears (Supplemental Figure 6, C–F). Moreover, under noise-free conditions, we compared hearing before and 7 days after *Kcnh8* delivery in 1-month-old mice and observed no difference (Supplemental Figure 6, G and H). We then subjected the WT mice to a 2-hour exposure of 8–16 kHz narrow-band noise at 97 dB SPL on day 6 after AAV injection, using the noninjected contralateral ears as internal controls. ABR assessment revealed that AAV-mediated *Kcnh8* delivery alleviated noise-induced hearing loss (Figure 6D). Notably, on postnoise day 1, the treated ears showed significant threshold improvements of 36 ± 2.6 dB (16 kHz), 26.5 ± 5.05 dB (24 kHz), and 16.5 ± 4.52 dB (32 kHz) over the contralateral controls. This protective effect persisted throughout the 14-day recovery period, as treated ears maintained lower thresholds by 16.11 ± 5.58 dB (16 kHz), 17.78 ± 5.71 dB (24 kHz), and 22.22 ± 6.78 dB (32 kHz) on day 14. These results demonstrate that exogenous *Kcnh8* gene delivery effectively mitigates noise-induced hearing loss in mice.

We further evaluated the therapeutic efficacy on HC morphology. Throughout the postnoise recovery period, no significant difference was observed in the number of MYO7A^+^ HCs between groups (Figure 6E). In contrast, analysis of synaptic pairs revealed a marked increase in CtBP2/HOMER1-positive puncta in AAV-treated ears compared with contralateral controls (Figure 6, E and F). Consistent with this synaptic preservation, TEM analysis demonstrated substantial ultrastructural improvement in treated HCs, characterized by more regularly organized vesicles and more abundant vesicles anchored at the synaptic ribbons (Figure 6, G and H). On day 7 after noise exposure, cochlear HCs were stained with MitoSOX and DCFH-DA to assess mitochondrial superoxide and intracellular ROS levels, respectively (Figure 6, I–L). The results showed that AAV-*Kcnh8* treatment significantly alleviated noise-induced HC damage. This protective effect corresponded with a marked reduction in noise-induced intracellular K^+^ accumulation (Figure 6, M and N). Collectively, these findings demonstrate that targeting *Kcnh8* effectively prevents noise-induced HC and synaptic degeneration, thereby preserving auditory function.

## Discussion

In this study, we demonstrate that *Kcnh8*, which encodes the voltage-gated potassium channel Kv12.1, is specifically expressed in cochlear HCs and is essential for maintaining intracellular potassium homeostasis (Figure 7). Notably, the in vivo function of *Kcnh8* had not previously been investigated in any mammalian system to our knowledge. Our work addresses this fundamental gap by establishing that *Kcnh8* deficiency leads to progressive hearing loss in mice, thereby revealing *Kcnh8*’s critical role in the auditory system. Mechanistically, we show that loss of *Kcnh8* impairs potassium efflux, resulting in HC hyperexcitability and ultimately leading to synaptic vesicle dysfunction. Furthermore, we found that targeted delivery of a *Kcnh8*-based construct ameliorates noise-induced HC damage and hearing loss by mitigating intracellular potassium accumulation. Collectively, our findings not only fill a key knowledge gap regarding the *KCNH* family in auditory research but also identify *Kcnh8* as a potential therapeutic target for the treatment of noise-induced hearing loss.

Notably, *Kcnh8* is the sole KCNH family gene we found to be specifically and highly expressed in both adult mouse IHCs and OHCs. Its high conservation and expression in human fetal HCs provide the necessary foundation for its function. This unique expression profile thus positions *Kcnh8* as a prime functional candidate representing the entire KCNH family in auditory processing. Prior evidence, though limited, has implicated *Kcnh8* in the excitability of thalamic neurons (32). A key in vitro study demonstrated that *Kcnh8* regulates the daytime/nighttime rhythms in repetitive firing rates of suprachiasmatic nucleus neurons (16). Despite its known roles, no study has directly linked *Kcnh8* to an organism-level phenotype through in vivo evidence. Our research fills this void by employing genetic intervention to definitively establish its requirement in maintaining auditory function.

We have identified *Kcnh8*-encoded Kv12.1 as a mediator of outward K^+^ currents in cochlear HCs, thereby exerting a functionally opposing effect to the excitatory K^+^ influx mediated by the nonselective mechanotransduction channel (33, 34). HCs act as the units for mechanoelectrical transduction; consequently, their excitability is closely linked to the function of K^+^ channels (35, 36). Previous studies have highlighted the crucial role of several outward K^+^ channels, with the most prominent examples being Kv7.4 (encoded by *Kcnq4*) (35, 37) and BK channels (38, 39). In terms of expression pattern, *Kcnq4* is specific to OHCs (37), while the BK channels are predominantly expressed in IHCs (40, 41). In contrast, *Kcnh8* is abundantly expressed in both IHCs and OHCs, distinguishing it from both. The distinct expression patterns and functions of these 3 types of outward K^+^ channels underlie their differential contributions to auditory function. By serving as a major determinant of the OHC resting membrane potential, the voltage-gated Kv7.4 channel mediates a noninactivating, slow, linear K^+^ current that is critical for K^+^ efflux (37, 42). Mutations in *KCNQ4* impair this efflux, resulting in chronic K^+^ overload, which in turn causes the severe, early-onset hereditary deafness observed in patients (19, 37). The loss of *Kcnh8* results in a progressive hearing impairment in mice, with a phenotypic similarity to that caused by BK channel dysfunction (43). The rapid repolarization mediated by BK channels, enabled by their fast activation kinetics and large conductance, is essential for tracking high-frequency stimuli (44, 45). Kv12.1 in IHCs operates on a different timescale. It mediates a component of the slowly activating I_K, s_ with intermediate kinetics, which contributes substantially to the outward conductance (26). Rather than driving the most rapid repolarization phases, this activity helps control the membrane potential during sound stimulation, thereby regulating IHC excitability. We found that *Kcnh8* deletion causes abnormal evoked action potentials and markedly increased IHC excitability at an early stage. Thus, we identify *Kcnh8* as a richly expressed K^+^ channel in both IHCs and OHCs, establishing its function as a key regulator of HC excitability.

We reveal that *Kcnh8* deficiency causes HC hyperexcitability at P10 because of impaired K^+^ efflux, paralleling the pathogenic excitability caused by other KCNH family members, such as *KCNH2* variants in long-QT syndrome 2 and epilepsy (46). By 1 M of age, this auditory function declines, suggesting that sustained hyperexcitability ultimately leads to functional loss, much like the hyperexcitability in *Kcnh7*-deficient Purkinje cells that causes behavioral deficits (10). The progressive delayed hearing loss by *Kcnh8* deficiency with advancing age might be partly attributed to a chronic intracellular K^+^ overload in the HCs, which may trigger a series of stress-induced injury responses. On the other hand, increased excitability upon acoustic stimulation led to excessive glutamate release, which may be responsible for part of the synaptic damage and hearing loss outcome. Similarly, neuronal hyperexcitability is crucially linked to neurodegeneration and synaptic abnormalities (6, 7), and excessive glutamate release is implicated in the pathogenesis of synapse damage and neurological disorders (8). Our findings suggest that impairment of the Kv12.1 channel causes progressive auditory decline. However, the mechanism by which intracellular K^+^ overload and glutamate excitotoxicity lead to hair cell degeneration requires further investigation.

The role of *Kcnh8* in maintaining K^+^ homeostasis in HCs provides a solid rationale and identifies it as a promising therapeutic target for treating hearing loss caused by environmental insults. Recent studies have shown that BK channel deficiency or pharmacological blockade ameliorates noise-induced HC degeneration (47, 48). Other reports conflictingly suggest that the lack of BK channels could instead increase noise vulnerability (24, 49). Our results provide compelling evidence that AAV-mediated delivery of *Kcnh8* mitigates noise-induced IHC damage by facilitating potassium efflux. In future studies, we will employ mouse models of noise-induced injury to both IHCs and OHCs at different intensities to evaluate *Kcnh8* effects on OHCs and its overall protective role. Notably, this gene delivery strategy proved effective in mice without employing a specific channel agonist, as none is currently available. This limitation stems from the unresolved protein structure of Kv12.1. Like other KCNH channels, Kv12.1 features intracellular N- and C-terminal regions containing a cyclic nucleotide-binding homology domain (50). These domains, serving as potential hubs for posttranslational modifications and molecular interactions, provide key structural insights. This knowledge thereby guides our future research aimed at solving the Kv12.1 structure, with the ultimate goal of developing specific small-molecule agonists.

In summary, we have identified Kv12.1 as a K^+^ channel highly enriched in auditory HCs and vital for auditory function. We further show that its role in K^+^ homeostasis is so critical that its restoration via gene delivery can alleviate noise-induced hearing damage. This work, therefore, progresses from the discovery of a fundamental auditory mechanism to the proposal of a viable therapeutic strategy, highlighting *Kcnh8* as a highly promising target in the fight against sensorineural hearing loss.

## Methods

### Sex as a biological variable.

Our study examined male and female animals, and similar findings are reported for both sexes.

### Generation of Kcnh8-KO mice via CRISPR/Cas9.

All mouse lines were maintained on a C57BL/6JGpt background, which holds the strain-specific hypomorphic *Cdh23*^ahl^ allele (51, 52). The *Kcnh8*-KO mice were generated by GemPharmatech Co., Ltd. using CRISPR/Cas9-mediated gene editing. Briefly, sgRNA was designed to target exon 4 of the *Kcnh8*-201 transcript (ENSMUST00000039366.11), synthesized in vitro, and coinjected with Cas9 protein into C57BL/6JGpt fertilized eggs. The microinjected embryos were then transferred to surrogate mothers to produce founder (F0) mice, which were genotyped by PCR and Sanger sequencing. A stable F1 line was established by crossing PCR-confirmed F0 mice with WT C57BL/6JGpt mice. Besides, Rosa26-tdTomato reporter mice were purchased from The Jackson Laboratory (Stock 007914).

### Human cochlea acquisition.

For the human component, cochlear samples were obtained from 2 medically aborted fetuses at 11 and 17 PCW. Cases involving severe pregnancy-related complications or congenital fetal abnormalities were excluded. All patient and sample data were anonymized and assigned numerical identifiers for confidentiality.

### Cochlear section and RNAscope in situ hybridization.

Human and mouse cochleae were rapidly dissected in phosphate-buffered saline (PBS). Mice were anesthetized with 0.4% Zoletil 50 (Virbac) and euthanized by cervical dislocation before dissection. Tissues were fixed overnight at 4°C in 4% paraformaldehyde (PFA; Sigma-Aldrich) in PBS, decalcified in 10% EDTA (SIMUWU bio) for 24 hours at room temperature (RT), and cryoprotected by immersion in a graded sucrose series (15% and 30% in PBS). Samples were embedded in Optimal Cutting Temperature compound (Sakura) and frozen on dry ice. Serial 12 μm cryosections were collected on SuperFrost Plus slides (Thermo Fisher Scientific), briefly warmed for adhesion, and stored at –20°C. Before hybridization, sections were postfixed in cold 4% PFA for 15 minutes, dehydrated through an ethanol series, air-dried, and encircled with a hydrophobic barrier (ImmEdge PAP pen, Vector Labs). To analyze mRNA spatial distribution in cochlear tissues, we performed RNAscope in situ hybridization (Advanced Cell Diagnostics) following manufacturer protocols. After the hydrophobic barriers dried, sections were digested with Protease IV and hybridized with target-specific probes (mouse *Kcnh8* or human *KCNH8*; Supplemental Table 2) for 2 hours at 40°C in a HybEZ oven. After hybridization, stringent washes were performed, followed by a series of signal amplification steps (AMP 1–4). The same sections were then processed for immunohistochemistry to enable multimodal analysis. Then, samples were counterstained with DAPI (5 μg/mL, 5 minutes, RT) and mounted for imaging. Confocal images were acquired using a Leica SP8 system and analyzed with Fiji software (Supplemental Table 5).

### ABR and DPOAE test.

Following intraperitoneal anesthesia (A mixture of 4 mg/mL Zoletil 50 and 5 μg/mL dexmedetomidine hydrochloride in saline), WT, *Kcnh8^+/–^*, and *Kcnh8^–/–^* mice from different age groups were maintained on a thermoregulated pad to keep warm. Subdermal needle electrodes (Rochester Electro-Medical) were positioned at standard recording sites. Tone burst stimuli (3 ms duration, 1 ms rise/fall) were generated using RZ6 system and BioSigRZ software (Tucker-Davis Technologies) and presented at 20/s. The testing paradigm employed 5 frequencies (4–32 kHz, octave steps) with intensity ranging from 90 to 0 dB SPL in 5 dB decrements. ABR thresholds were confirmed by visual examination as the lowest stimulus level in which a repeatable waveform could be observed. DPOAE recordings were obtained for primary tones with a frequency ratio (f2/f1) of 1.2 and a level ratio of L1 = L2 + 10 dB SPL using RZ6 system and BioSigRZ software (Tucker-Davis Technologies). The f2 frequency was varied from 4 to 32 kHz in half-octave steps. The level of the primary tones was swept in 5 dB SPL steps from 80 to 10 dB SPL (re: f2). The DPOAE threshold was defined as the f1 level required to elicit a distortion product of 5 dB SPL, which was determined from the averaged spectra. All measurements were conducted using the same device and by the same operator to ensure consistency.

### AAV delivery.

Mice were anesthetized by intraperitoneal injection of a mixture of 4 mg/mL Zoletil 50 and 5 μg/mL dexmedetomidine hydrochloride (DEXDOMITOR) in sterile saline, with body temperature maintained using a heating pad throughout the procedure. A small incision was made underneath the pinna using microsurgical scissors, followed by careful tissue dissection to expose the tympanic bulla. The round window membrane was identified under direct visualization, and microinjections were performed using a stereomicroscope (OLYMPUS) coupled with an MX160 micromanipulator (WPI). AAV delivery was achieved via a glass micropipette (10–15 μm tip diameter) connected to a Nanoliter 2000 microinjection system (WPI), with a controlled infusion rate of 50 nL/min. A total volume of 1–2 μL of AAV suspension (at the maximum achievable titer) was delivered into the cochlea. The surgical site was closed with interrupted 6-0 Vicryl sutures (Ningbo Medical Needle), followed by standard postoperative care that included analgesia and daily monitoring.

### Counting.

For synaptic pair counting, paired CtBP2 and HOMER1 signals were counted. The boundary of each IHC was outlined based on MYO7A labeling to define the counting region. For each field of view, a *z*-stack was captured at a 1 μm step size and projected as maximum-intensity projections using LAS X (Leica) software. Images were analyzed and reconstructed in Fiji (v2.9.0) based on MYO7A labeling to obtain the full IHC outline. For TEM, the electron-dense core of the ribbon was used as the center, and vesicles within 100 nm of its surface were classified as ribbon-associated vesicles. Free vesicles were defined as vesicles within 1 μm of the presynaptic membrane, excluding ribbon-associated vesicles. Synaptic puncta (CtBP2/HOMER1 pairs) and vesicles were then counted manually in ImageJ. All counts were performed in a blinded manner: each sample was counted before group assignment. 

### Statistics.

All statistical analyses and graphing were performed using GraphPad Prism (v9.3.1). For comparisons between 2 groups, an unpaired 2-tailed Student’s *t* test was used. For comparisons across multiple groups, 1-way ANOVA was applied, followed by either Dunnett’s multiple-comparison test (when comparing all groups with a single control) or Tukey’s multiple-comparison test (for comparisons against every other group). A *P* value of less than 0.05 was considered statistically significant. Data are presented as mean ± SEM.

### Study approval.

For the animal study, all mice were housed in the Fudan University Animal Facility under controlled conditions: 22°C ± 1°C ambient temperature, 30%–70% humidity, and a 12-hour light/12-hour dark cycle. All experimental procedures of mice were approved by the Fudan University Institutional Animal Care and Use Committee. For the human study, informed consent was obtained from the pregnant women after a comprehensive explanation of the study’s use of fetal tissues. The research protocol received ethical approval from the Ethics Committee of the Eye & ENT Hospital of Fudan University, China (Project No. 2022166).

### Data availability.

Two public datasets were used in this study. The adult mouse cochlea snRNA-seq dataset was extracted from Gene Expression Omnibus database (GSE274279). The postnatal mouse cochlea scRNA-seq dataset was extracted from Array Express (EMTAB14691). Other methods including genotyping of *Kcnh8*-KO mice, qRT-PCR, Western blot, immunohistochemistry, HiUGE system AAV design and tissue culture, therapeutic AAV design and packaging, SEM and TEM, intracellular potassium ion measurement, oxidative stress detection, and electrophysiological analysis are detailed in Supplemental Methods file. Detailed values of graph data in both the main text and supplemental material are available in the Supporting Data Values file. Additional datasets generated and analyzed during the current study are available from the corresponding author (WL) upon reasonable request.

## Manuscript information

### Author contributions.

WL, HL, and MX conceptualized the study. WL, MX, YJ, Y Liu, JM, XG, and Y Ding contributed to the methodology. Y Liu, YJ, JM, XG, Y Li, Y Dong, MW, GJ, YH, Y Lou, MX, and TS contributed to the investigation. Y Liu, YJ, and MX contributed to the visualization of data. WL, HL, MX, and YJ contributed to the funding acquisition and resources. WL, HL, and MX administrated and supervised the project. Y Liu, YJ, and MX wrote the original draft of the manuscript, and WL, HL, MX, YJ, and Y Liu reviewed and edited the manuscript. Y Liu and YJ contributed equally and are listed according to their relative contributions.

### AI disclosure.

We used DeepSeek and Grammarly to assist with grammar checking.

### Conflict of interest.

The authors have declared that no conflict of interest exists.

### Funding.

This study was supported by the following:

- STI2030-Major Projects, 2022ZD0205400, to WL.
- National Natural Science Foundation of China, 82425018, 82192865, and 82271170 to WL, 82192861, to HL, 82471189, to MX.
- National Key R&D Program of China, 2023YFC2508000, to HL.
- Natural Science Foundation of Shanghai, 25ZR1402060, to YJ.
- Shanghai “Rising Stars of Medical Talents” Youth Development Program, SHWSRS (2025) 071, to MX.

## Supplementary Material

Supplemental data

Unedited blot and gel images

Supporting data values

## Figures and Tables

**Figure 1:**
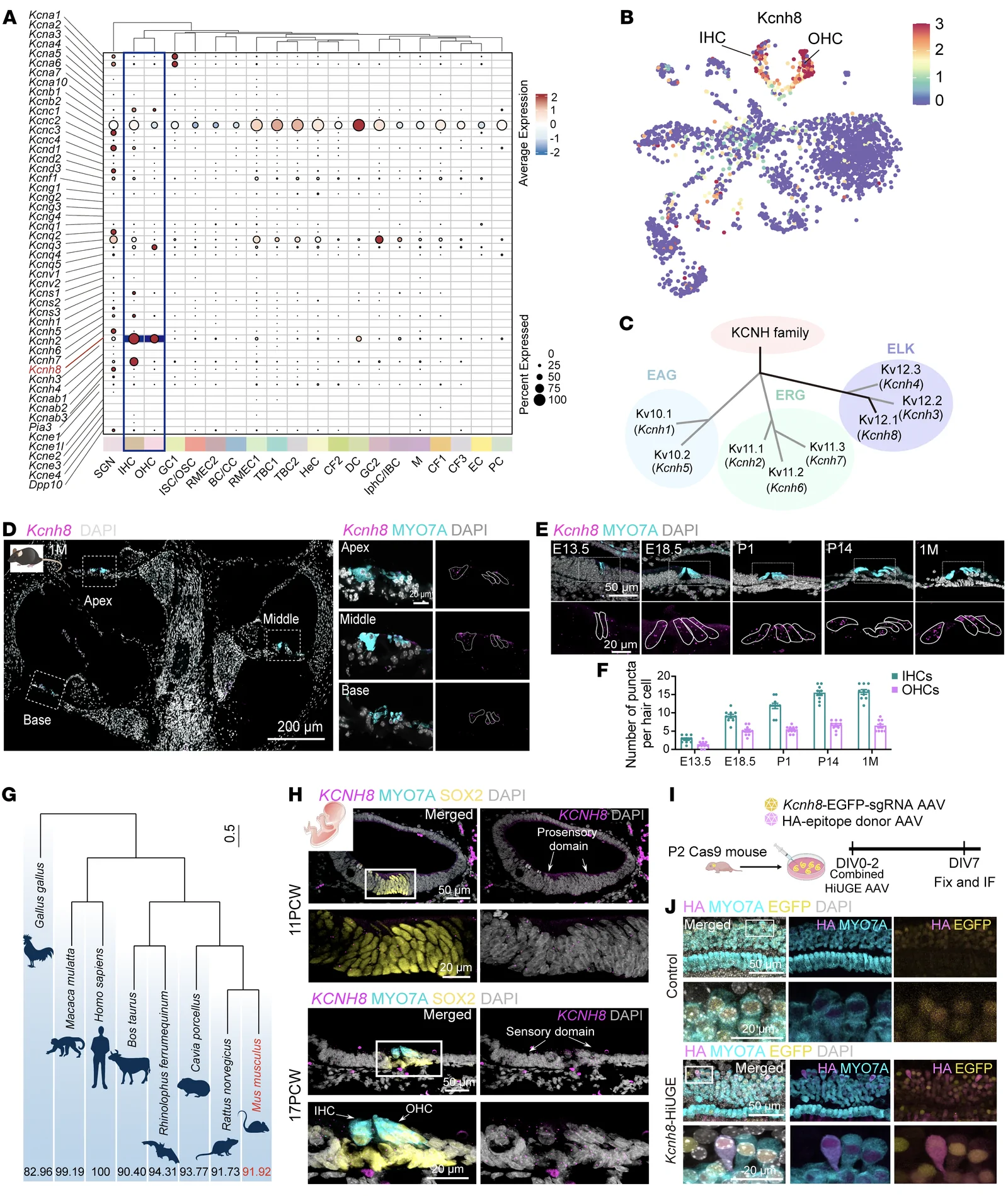
Expression and subcellular localization of *Kcnh8* (Kv12.1) in the mouse and human cochlea. (**A**) Dot plot depicting the expression of all KCNH family members across distinct cell types in the adult mouse cochlea. (**B**) Feature plot illustrating the restricted expression of *Kcnh8* within IHCs and OHCs. (**C**) Schematic of the KCNH channel family, highlighting the classification of Kv12.1 (encoded by *Kcnh8*) within the ELK subfamily. (**D**) Spatial localization of *Kcnh8* RNA (shown in magenta) in mouse cochlea at 1 M, as detected by RNAscope. Gray dashed lines outline IHCs and OHCs. (**E**) Developmental time course of *Kcnh8* RNA (magenta) expression in the mouse cochlea at E13.5, E18.5, P1, P14, and 1 M. Sections were costained for HC marker MYO7A (cyan) and DAPI (nuclei, gray). (**F**) Quantification of *Kcnh8* RNA puncta per IHC (green) and OHC (magenta) from the experiments shown in **E**. (**G**) Phylogenetic analysis of *Kcnh8* from representative species, based on amino acid sequence alignment. Percentage identity to the human protein is indicated. (**H**) Expression of *Kcnh8* RNA in human fetal cochlea at PCW 11 and 17. Left panels show merged channels (*Kcnh8*, magenta; MYO7A, cyan; SOX2, yellow; DAPI, gray); right panels show colabeling of *Kcnh8* and DAPI. Upper panels, low-magnification views with white boxes indicating regions of interest; lower panels, higher magnification views of individual channels. (**I**) Schematic of the HiUGE strategy for knockin of an HA-epitope tag to label the Kv12.1 (*Kcnh8*-encoding protein) in vitro. DIV, days in vitro; IF, immunofluorescence. (**J**) Representative confocal images showing HA-tagged Kv12.1 following AAV transduction. EGFP indicates transfected cells. Left panels show merged channels (HA, magenta; MYO7A, cyan; EGFP, yellow; DAPI, gray); middle panels show colabeling of HA and MYO7A; right panels show colabeling of HA and EGFP. Scale bars are provided in **D**, **E**, **H**, and **J**.

**Figure 2:**
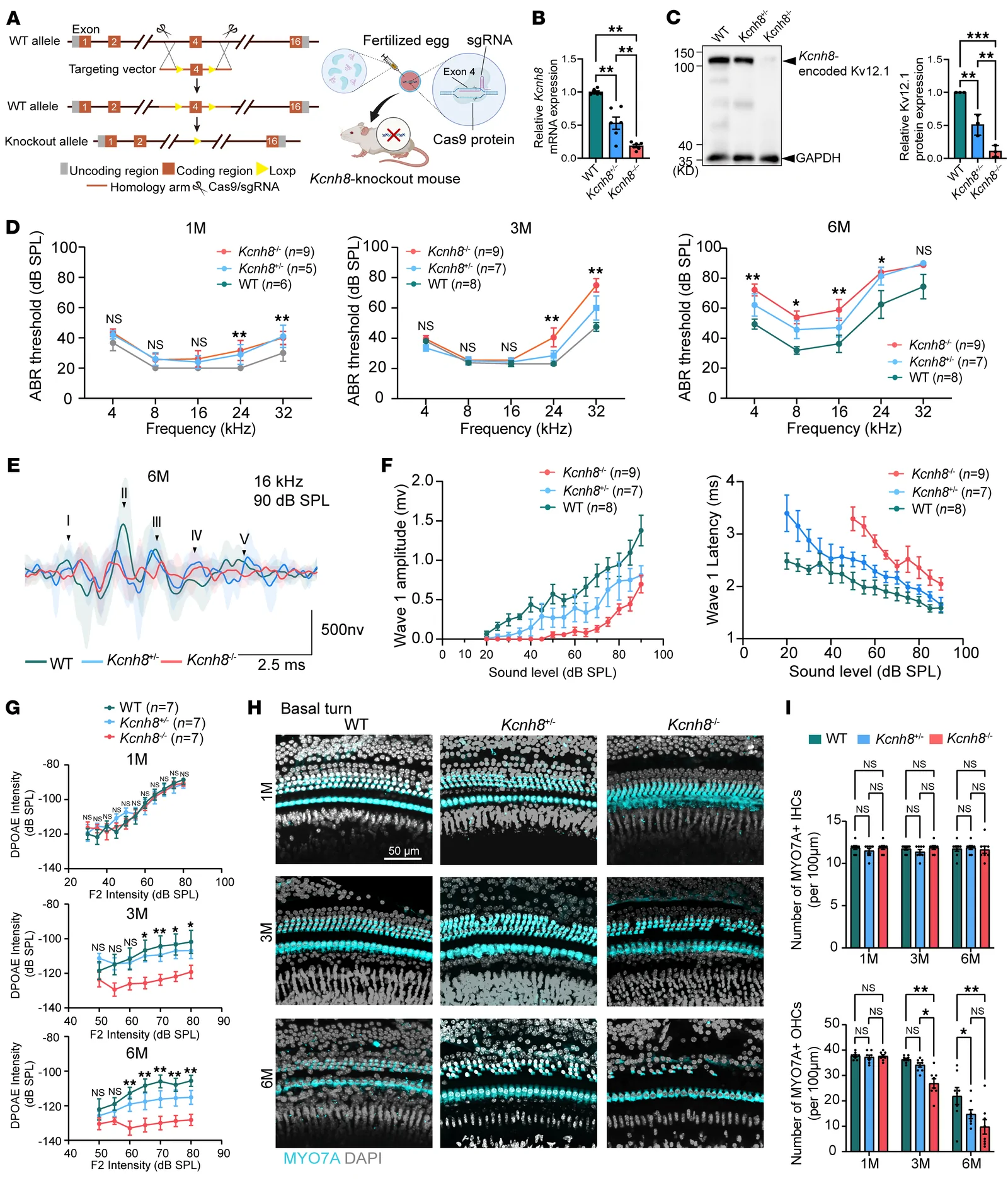
Progressive hearing loss in the absence of *Kcnh8*. (**A**) Strategy for generating *Kcnh8*-knockout mice via CRISPR/Cas9. Exon 4 of the *Kcnh8* gene was deleted to generate a null allele. (**B** and **C**) Validation of *Kcnh8* knockout at RNA and protein levels. (**B**) qRT-PCR and (**C**) Western blot analysis of *Kcnh8* transcript and protein expression in WT, *Kcnh8^+/–^*, and *Kcnh8*^–/–^ mice. GAPDH served as the internal control. (**D**) ABR thresholds across 4–32 kHz frequencies in WT, *Kcnh8^+/–^*, and *Kcnh8*^–/–^ mice at 1, 3, and 6 M. (**E**) ABR waveforms evoked by a 32 kHz tone at 90 dB sound pressure level (SPL) of 3 M WT, *Kcnh8^+/–^*, and *Kcnh8*^–/–^ mice. Arrowheads indicate peaks I–V. (**F**) Quantification of wave I peak amplitude (left) and latency (right) from the recordings in **E**. (**G**) DPOAE intensity in WT and *Kcnh8*^–/–^ mice at 1, 3, and 6 M. (**H**) Representative confocal images of the basal cochlear turn from WT and *Kcnh8*^–/–^ mice at 1, 3, and 6 M, immunolabeled for Myosin7a (MYO7A) (cyan) and DAPI (nuclei, gray). Scale bar, 50 μm. (**I**) Quantification of MYO7A-positive IHCs (upper panel) and OHCs (lower panel) per 100 μm cochlear length from the samples shown in **H**. *n* = 8. Statistical significance was determined using 2-way ANOVA with Tukey’s post hoc test or unpaired 2-tailed *t* test, as appropriate (*P < 0.05, **P < 0.01). The number (*n*) of mice was indicated in each group.

**Figure 3:**
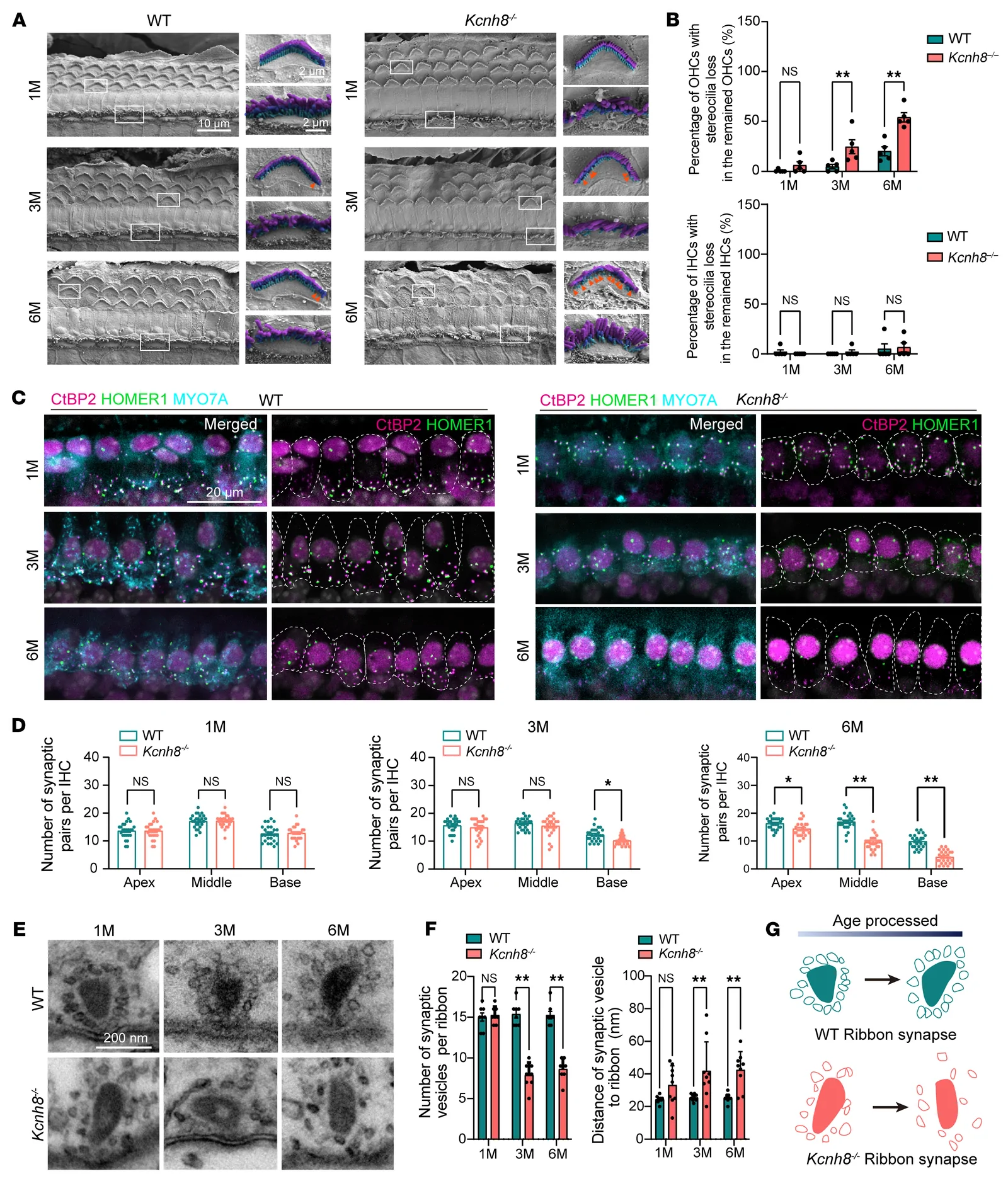
Stereocilia loss and ribbon synapse degeneration in *Kcnh8*-deficient HCs. (**A**) SEM images of the cochlear sensory epithelium (left) and magnified views of stereocilia bundles on IHCs and OHCs (right) from the basal turn of WT and *Kcnh8*^–/–^ mice at 1, 3, and 6 M. The 3 rows of OHC stereocilia are color-coded. Red arrowheads indicate missing stereocilia. (**B**) Quantification of the percentage of stereocilia loss in OHCs (upper panel) and IHCs (lower panel) in WT and *Kcnh8*^–/–^ mice (*n* = 5) from images in **A**. (**C**) CtBP2 (presynaptic ribbons, magenta), HOMER1 (postsynaptic terminals, green), MYO7A (HCs, cyan), and DAPI (nuclei, gray). Left panels show merged channels; right panels show CtBP2 and HOMER1 colabeling. IHC outlines are demarcated by gray dashed lines. (**D**) Quantification of the number of synaptic pairs per IHC from the apex, middle, and basal turns in WT and *Kcnh8*^–/–^ mice. *n* = 25 IHCs per group from 5 mice. (**E**) Representative TEM images of IHC ribbon synapses of the basal turn from WT and *Kcnh8*^–/–^ mice at 1, 3, and 6 M. (**F**) Quantification of the number of synaptic vesicles per ribbon (left panel) and the distance from the synaptic vesicle to ribbon (right panel) in IHCs of WT and *Kcnh8*^–/–^ mice from images in **E**. *n* = 8 ribbon synapses from 5 mice for each group. (**G**) Schematic model summarizing the progressive structural deterioration of IHC ribbon synapses in *Kcnh8*^–/–^ mice from 1 to 6 M. Statistical significance was determined using 2-way ANOVA with Tukey’s post hoc test, as appropriate (*P < 0.05, **P < 0.01). Scale bars are provided in **A**, **C**, and **E**.

**Figure 4:**
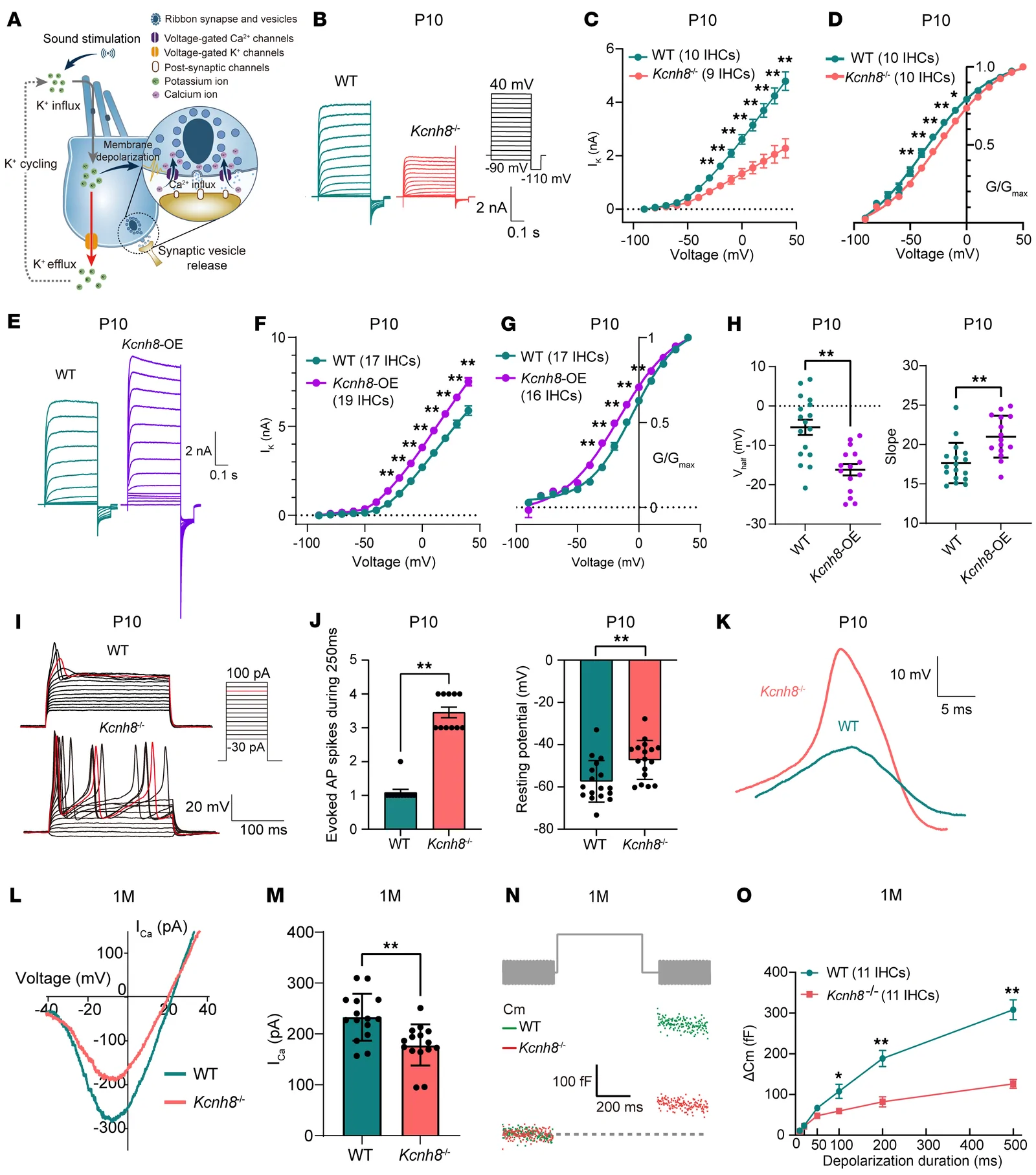
*Kcnh8* regulates IHC excitability and synaptic transmission. (**A**) Model of HC auditory transduction and the K^+^-initiated cascade. (**B**) Representative whole-cell K^+^ currents recorded from P10 WT and *Kcnh8*^–/–^ IHCs. Currents were elicited by stepping from a holding potential of –80 mV to test potentials ranging from –90 mV to +40 mV, followed by a step to –110 mV. (**C**) Steady-state K^+^ currents (I_K_) from P10 WT and *Kcnh8*^–/–^ IHCs from 5 mice. (**D**) Voltage dependence of activation derived from currents in **B**. Solid lines indicate Boltzmann fits. (**E**) Representative whole-cell K^+^ currents from P10 WT and *Kcnh8*-overexpressing (*Kcnh8*-OE) IHCs using the same protocol as in **B**. (**F**) Steady-state K^+^ currents from P10 WT and *Kcnh8*-OE IHCs from 5 mice. (**G**) Activation curves and Boltzmann fits from WT and *Kcnh8*-OE recordings. (**H**) Half-activation voltage (*V_half_*, left) and slope factor (right) derived from fits in **G**. (**I**) Representative current-evoked action potentials (APs) recorded from P10 WT (top) and *Kcnh8*^–/–^ (bottom) IHCs. The 80 pA trace is highlighted in red. (**J**) Quantification of the number of evoked APs during a 250 ms current injection and resting membrane potential from P10 WT and *Kcnh8*^–/–^ IHCs (*n* = 5 mice). (**K**) Representative single AP waveforms extracted from the 80 pA injection traces in **I**. (**L**) Representative I–V curves of Ca^2+^ currents recorded from IHCs of WT and *Kcnh8*^–/–^ mice at 1 month old. Currents were evoked by a voltage ramp from –90 to +70 mV under voltage clamp and subsequently leak-subtracted. (**M**) Comparison of the peak Ca^2+^ current from 1-month-old WT and *Kcnh8*^–/–^ IHCs from 8 mice. (**N**) A representative diagram of I_Ca_ and the corresponding changes in membrane capacitance (ΔCm) in WT and *Kcnh8*^–/–^ IHCs at 1 M of stimulation of duration 500 ms. fF, femtofarads. (**O**) ΔCm, reflecting vesicle exocytosis, evoked by depolarizing pulses (10–500 ms) in 1 M WT and *Kcnh8*^–/–^ IHCs from 8 mice. Data are reported as mean ± SEM. Statistical significance was determined by unpaired 2-tailed *t* test or 2-way ANOVA with Tukey’s post hoc test (*P < 0.05, **P < 0.01). The number of patched IHCs was indicated in each group.

**Figure 5:**
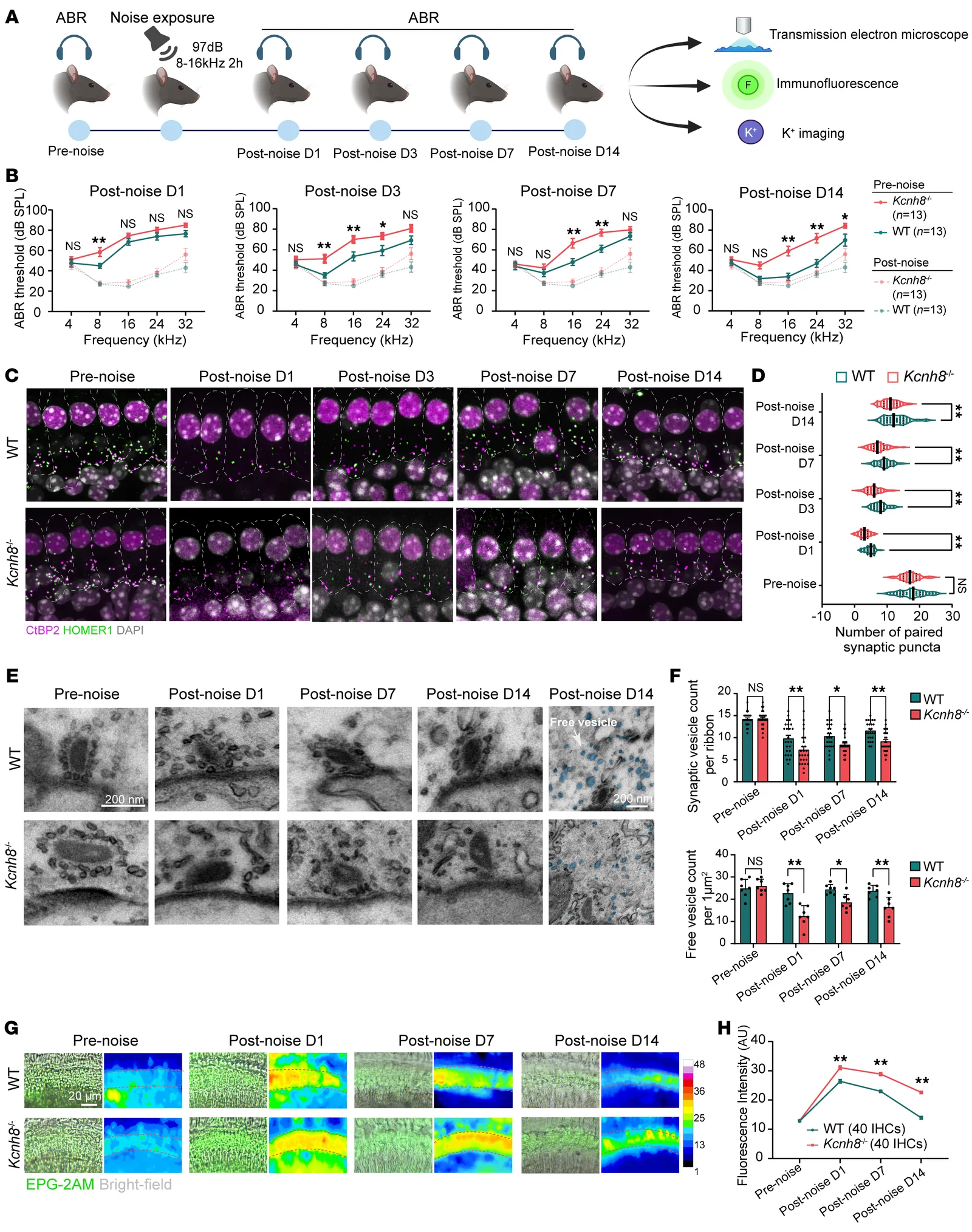
*Kcnh8* deficiency exacerbates the susceptibility to noise exposure. (**A**) Schematic of the noise exposure experimental workflow. (**B**) ABR threshold in WT and *Kcnh8*^–/–^ mice before and after noise exposure. Dashed lines indicate prenoise thresholds for each genotype. (**C**) Representative confocal images of IHC ribbon synapses from the middle turns of WT and *Kcnh8*^–/–^ mice before and after noise exposure. Tissues were immunolabeled for CtBP2 (magenta), HOMER1 (green), and DAPI (nuclei, gray). (**D**) Quantification of the number of synaptic pairs per IHC from images in **C**. 50 IHCs from 8 mice per group. (**E**) Representative TEM images of IHC ribbon synapses from the middle turns of WT and *Kcnh8*^–/–^ mice before and after noise exposure. White arrows indicate free vesicles at postnoise day 14. (**F**) Quantification of the number of synaptic vesicles tethered to each ribbon (upper panels) and the number of free vesicles in the surrounding 1 μm^2^ area of ribbons (lower panel) from the TEM images in **E**. 25 ribbons from 7 mice per group. (**G**) Representative fluorescence and pseudocolor images showing intracellular K^+^ concentration in IHCs of the middle turns of WT and *Kcnh8*^–/–^ mice, measured using the EPG-2AM K^+^ indicator. IHCs are outlined by red dashed lines. (**H**) Quantification of the mean fluorescence intensity of EPG-2AM in IHCs from WT and *Kcnh8*^–/–^ mice (*n* = 5) before and after noise exposure from images in **G**. Data are presented as means ± SEMs. Statistical significance was determined using 2-way ANOVA with Tukey’s post hoc test or unpaired 2-tailed *t* test, as appropriate (*P < 0.05, **P < 0.01). Scale bars are provided in **C**, **E**, and **G**.

**Figure 6:**
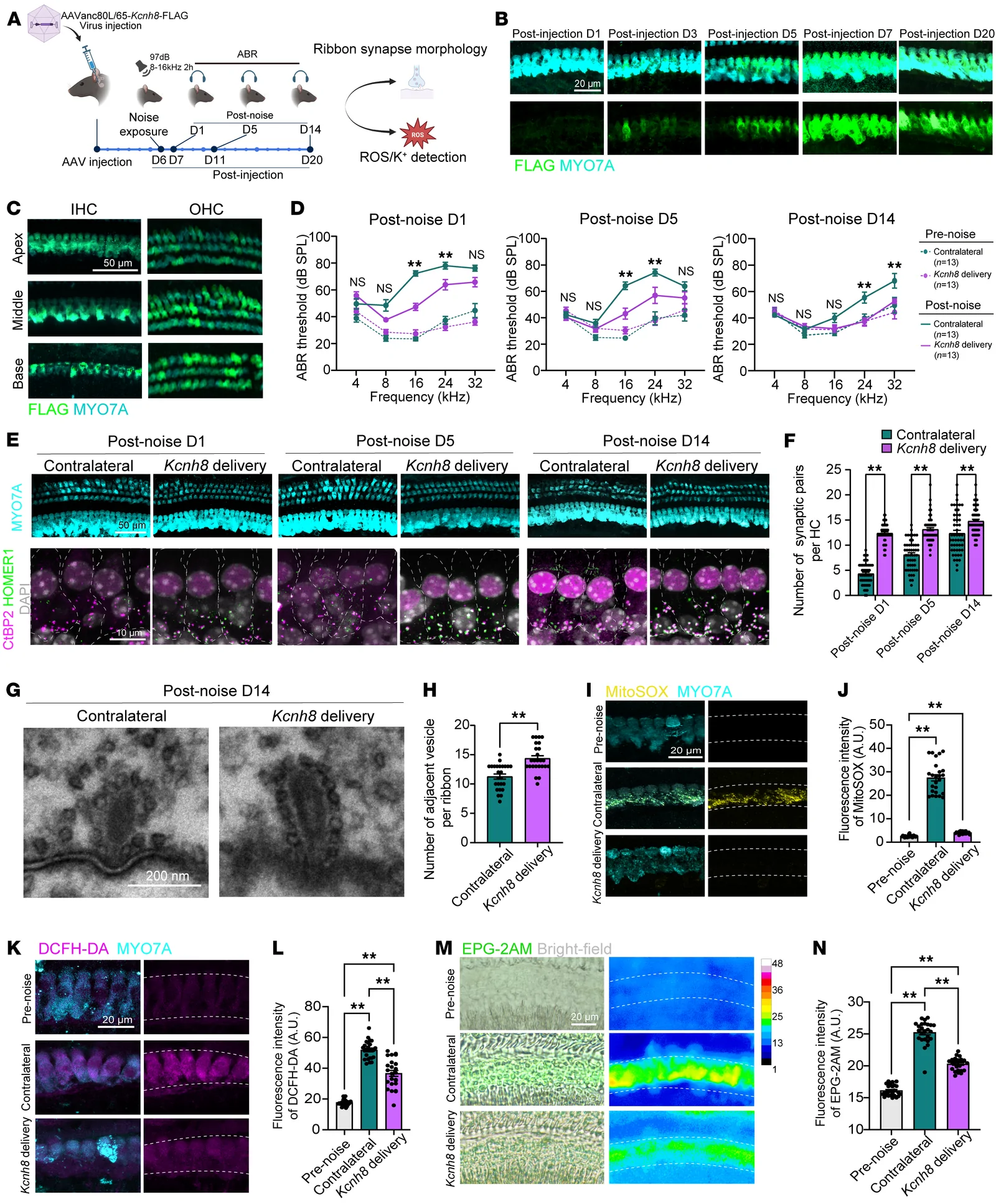
AAV-mediated *Kcnh8* overexpression protects against noise-induced damage in WT mice. (**A**) Schematic diagram of the experimental timeline for noise exposure and AAV delivery. (**B**) Time course of AAV-*Kcnh8* transduction efficiency in the middle turn of the cochlea, assessed by FLAG (*Kcnh8*-encoding protein, green) and MYO7A (cyan) colabeling. (**C**) Representative confocal images showing successful transduction of AAV-*Kcnh8* (FLAG, green) in HCs throughout the apex, middle, and base turns of the cochlea. (**D**) ABR threshold from AAV-*Kcnh8*–treated ears and contralateral ears after noise exposure days 1, 5, and 14 (*n* = 13). Dashed lines indicate prenoise thresholds for treated and contralateral ears. (**E**) Representative confocal images of IHC ribbon synapses in the middle turns of treated and contralateral ears at postnoise days 1, 5, and 14. Tissues were costained with MYO7A (cyan), CtBP2 (presynaptic ribbons, magenta), HOMER1 (green), and DAPI (gray). (**F**) Quantification of the number of synaptic pairs per IHC from the images in **E**. 50 IHCs from 5 mice per group. (**G**) Representative TEM images of IHC ribbon synapses in the middle turns of treated and contralateral ears at postnoise day 14. (**H**) Quantification of the number of synaptic vesicles per ribbon from the TEM images in **G**. 28 ribbons from 6 mice per group. (**I**–**L**) Representative confocal images of MitoSOX (yellow) and DCFH-DA (cyan) in IHCs of the middle turns of treated, contralateral, and prenoise ears at postnoise day 7. Fluorescence intensities of MitoSOX and DCFH-DA are shown in **J** and **L**. (**M**) Representative fluorescence images of intracellular K^+^ concentration, measured using the EPG-2AM indicator, in IHCs of the middle turns of treated, contralateral, and control ears at postnoise day 7. (**N**) Fluorescence intensity of EPG-2AM from the images in **M**. 28 IHCs from 6 mice per group in **I**, **L**, and **N**. Data are presented as means ± SEMs. Statistical significance was determined using 2-way ANOVA with Tukey’s post hoc test or unpaired 2-tailed *t* test (**P < 0.01).

**Figure 7:**
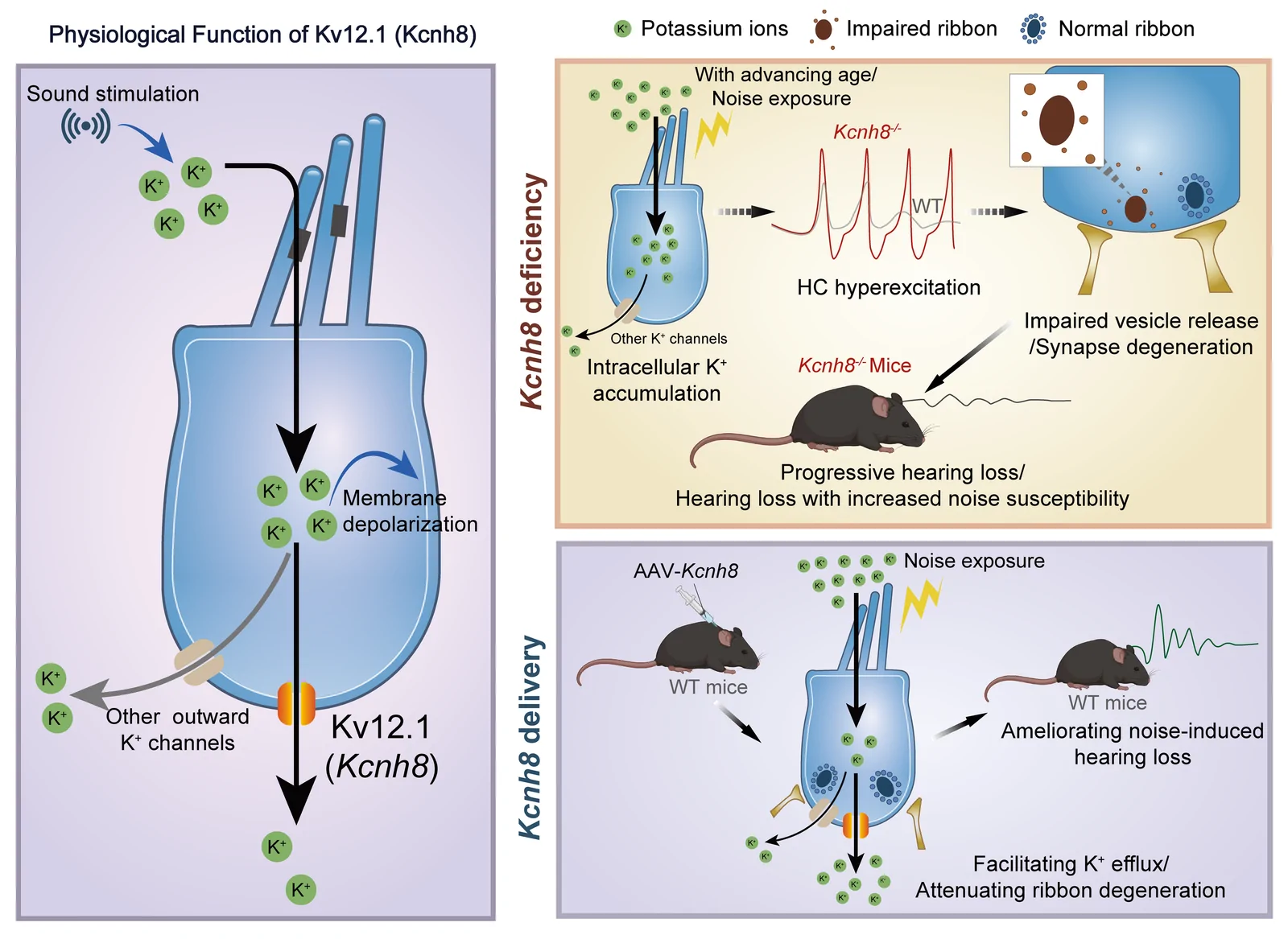
Role of the Kv12.1 (*Kcnh8*) K^+^ efflux channel in hearing and therapy for noise-induced loss. Under physiological conditions, *Kcnh8*-encoded Kv12.1 channel provides a crucial K^+^ efflux pathway that stabilizes the membrane potential of HCs during sound stimulation, ensuring precise synaptic transmission. *Kcnh8* deficiency disrupts K^+^ homeostasis, causing IHC hyperexcitability, excitotoxic synaptic damage, and progressive hearing loss — a pathology exacerbated by noise exposure. Conversely, in noise-exposed WT mice, AAV-mediated *Kcnh8* delivery into the cochlea promotes K^+^ efflux, prevents ribbon synapse degeneration, and accelerates hearing recovery, highlighting its therapeutic potential.

## Version 1 11/16/2026

Electronic publication
