# Supplementary material for: Kv12.1 (Kcnh8) maintains hair cell potassium homeostasis and protects against noise-induced hearing loss in mice
Source: J Clin Invest. 2026 Nov 16;136(22):e202988. doi: 10.1172/JCI202988 (PMC13767643; doi:10.1172/JCI202988)
Supplement: Supplementary file 1 [file jci-136-202988-s239.pdf]

Supplementary Material

Supplementary Methods

Genotyping of Kcnh8 KO mice. To genotype *Kcnh8* knockout mice, genomic DNA was extracted from tail biopsies and analyzed by PCR using two specific primer pairs. One pair (F1/R1) amplified a sequence encompassing exon 4, while the other (F2/R2) targeted an internal region of the same exon, allowing for definitive identification of the wild-type and mutant alleles. The wild-type (WT) allele was amplified to yield 2682 bp and 308 bp fragments, respectively. In KO mice, primer pair F1+R1 generated a shorter 481 amplicon and no production of primes F2+R2 was detected due to the targeted deletion. PCR products were resolved on 1.5% agarose gels (TIANGEN biotech) and visualized under UV light using a digital imaging system (INTAS Science Imaging Instruments). Primers for genotyping were as listed in Table S1.

Reverse transcription (RT)-PCR and quantitative reverse transcription (qRT)-PCR. Cochlear epithelial tissues were rapidly dissected from sacrificed mice and immediately snap-frozen in liquid nitrogen. For each experimental group, tissues from four mice (n=4) were pooled and homogenized using a cryogenic mortar and pestle. Total RNA was isolated from the homogenates using Trizol reagent (Invitrogen) followed by chloroform (Thermo Fisher Scientific) extraction and isopropanol precipitation, according to the manufacturer's instructions. RNA concentration and purity were determined using a NanoDrop ND-1000 spectrophotometer.

For RT-PCR, 2 μL of the resulting cDNA was amplified with Taq DNA Polymerase (Biomed) using gene-specific primers (Table S1). The PCR protocol consisted of an

initial denaturation at 94 °C for 5 min, followed by 35 cycles of 94 °C for 30 s, 60 °C for 45 s, and 72 °C for 60 s, with a final extension at 72 °C for 10 min. The PCR products were separated on 2% agarose gels stained with ethidium bromide and visualized under UV light. The band intensities for *Kcnh8* were quantified using ImageJ software (NIH) and normalized to those of *Gapdh*. Reverse transcription was performed using the PrimeScript™ RT Reagent Kit (Takara) at 42 °C for 50 minutes.

Quantitative PCR was carried out using Power SYBR Green PCR Master Mix on an CFX Opus 96 Real-Time PCR System (Bio-rad). Each 20 μL reaction contained 1× SYBR Green Master Mix, 0.4 μM of each primer, and 2 μL of a 1:10 dilution of cDNA. The thermal cycling conditions were: 50 °C for 2 min, 95 °C for 10 min, followed by 40 cycles of 95 °C for 15 s and 60 °C for 1 min. A melt curve analysis was performed post-amplification to confirm amplification specificity. All samples were run in technical triplicates. The *Gapdh* gene was used as an endogenous control for normalization, and the relative expression levels of *Kcnh8* were calculated using the $2^{(-\Delta\Delta Ct)}$ method.

Western Blot. Sufficient samples were dissected from cochlea were homogenized and incubated in mixtures of RIPA lysis buffer and 1×protease and phosphatase inhibitors cocktail for 30 minutes on ice. Subsequently, the lysates were centrifuged at 12,000 × g for 15 min at 4 °C. The supernatant was carefully aspirated, aliquoted, and stored at -80 °C until use. Protein concentration was determined using a Pierce BCA Protein Assay Kit with BSA standards prepared in the same RIPA buffer. Equal amounts of protein (40 μg) from each sample were mixed with 5×loading buffer, boiled at 95 °C for 5 min, and then separated on a 10% SDS-polyacrylamide gel. Immunoblotting. The separated proteins were electrophoretically transferred to a PVDF membrane activated in methanol. The membrane was blocked in 5% BSA in TBST for 1 h at room

temperature and then incubated with anti-Kv12.1 primary antibody (1:1000, Thermo Fisher Scientific) diluted in 5% BSA/TBST overnight at 4 °C. After three washes with TBST, the membrane was incubated with an HRP-conjugated goat anti-rabbit secondary antibody (1:2000, Thermo Fisher Scientific) and anti-GAPDH antibody HRP conjugated (1:20000, Signalway Antibody) for 1 h at room temperature. Signal detection was performed using SuperSignal West Pico PLUS Chemiluminescent Substrate (Thermo Fisher Scientific) and a Bio-Rad ChemiDoc MP Imaging System (Bio-Rad). Band intensities were quantified using ImageLab software (Bio-Rad).

Immunohistochemistry. Following extraction from the temporal bone in PBS, cochlear tissues were fixed in 4% PFA at 4 °C overnight. After PBS washes, specimens underwent decalcification in 10% EDTA (24 hours) before microdissection of the organ of Corti. Tissue blocks were permeabilized and blocked simultaneously in 0.5% Triton X-100 (Sigma-Aldrich) with 5% normal goat serum (Sangon Biotech) (1 hour, RT).

Primary antibody incubations were performed in 0.5% Triton dilution in PBS at 4°C overnight, followed by 4-hour RT incubation with species-matched secondary antibodies (Alexa Fluor 488/555/647 conjugates, 1:400 in 1% Triton/PBS). Antibodies were as listed in Table S4. Post-staining washes in PBS preceded nuclear counterstaining with DAPI. Confocal imaging was conducted using a Leica SP8 system, with subsequent analysis performed in Fiji/ImageJ.

Intracellular potassium ion measure. Intracellular potassium ions were measured using the fluorescent probe EPG-2AM (MX4513; Maokang Biotechnology), a potassium indicator that is cell-membrane permeable. Briefly, dissected cochlear tissues were incubated in DMEM culture medium containing 10 μM EPG-2AM for 60 minutes, followed by a 30-minute incubation in probe-free DMEM. The tissues were then

washed twice with PBS. Fluorescence images were acquired using a confocal laser scanning microscope, and the fluorescence intensity was quantified using ImageJ software.

*Detection of oxidative stress***.** Intracellular ROS and mitochondrial superoxide levels were evaluated using DCFH-DA (Invitrogen) and MitoSOX (Invitrogen), respectively. The organs of Corti were acutely dissected as described above and incubated with 2 μM MitoSOX and 2 μM DCFH-DA for 30 minutes at 37°C. Images were captured using a confocal laser scanning microscope. Subsequently, the IHCs of interest were outlined as regions of interest (ROIs), and their fluorescence intensity was quantified using ImageJ software.

Image Acquirement: All confocal images were acquired using a Leica TCS SP8 confocal microscope with either a 63×/1.4 NA or a 40×/1.15 NA oil-immersion objective. For each field of view, a z-stack with a step size of 1 μm was captured. These stacks were then converted into maximum intensity projections using LAS X (Leica) software. The same acquisition and projection parameters were applied consistently to all experimental groups.

HiUGE system AAV design and tissue culture. The HIUGE knock-in system was deployed based on an established protocol (50). Briefly, two AAV vectors were constructed: one expressing a *Kcnh8*-specific gRNA (*Kcnh8*-gRNA) and the other expressing a donor-specific gRNA (DS-gRNA). The DS-gRNA and *Kcnh8*-gRNA were designed to target a donor recognition sequence (DRS) and the specific genomic locus of *Kcnh8* (cacaagcacagcggatgtaaagg), respectively. The DRS facilitates the precise assembly and subsequent integration of the donor payload—which encodes an

HA tag—into the *Kcnh8* locus. This successful integration results in the expression of the endogenous Kcnh8 protein fused with the HA tag.

For *in vitro* culture, cochlear tissues isolated from P2 Rosa26 mice were cultured in DMEM/F12-based medium (Thermo Fisher Scientific) supplemented with B27 (Thermo Fisher Scientific) and N2 (Thermo Fisher Scientific). The explants were transduced with the HIUGE system's dual AAV vectors. After 48 hours post-transduction, the medium was changed, and the cultures were maintained until culture day 7. The samples were then processed for subsequent immunofluorescence staining.

Therapeutic AAV design and packaging. The AAV vector for *Kcnh8* overexpression was constructed to contain a cytomegalovirus (CMV) promoter, the murine Kcnh8 gene (RefSeq: NM_001031811.2), and a C-terminal 3×FLAG tag sequence. The corresponding *Kcnh8*-3×FLAG coding sequence was synthesized, amplified by PCR, and subsequently cloned into a pAAV2 vector. The integrity of the synthesized plasmid was verified through multiple restriction enzyme digestions and Sanger sequencing. The final transfer plasmid, pAAV-Anc80L65-CMV.m*Kcnh8*-3×FLAG-pA, along with the pAAV-Anc80L65 packaging plasmid, were procured from Shanghai Taitools Bioscience Co., Ltd.

Scanning electron microscopy (SEM). Cochlear tissues were collected from both WT and *Kcnh8*$^{-/-}$ mice at different stages. Immediately following dissection, specimens underwent primary fixation by immersion in 2.5% glutaraldehyde prepared in 0.1 M phosphate buffer for 2-3 hours at RT. After fixation, complete decalcification was achieved through incubation in 0.25 M EDTA solution at 4 °C for 24 hours. Tissues then received secondary fixation in 1% OsO4 for 60 minutes at RT. The organs of Corti were carefully dissected in PBS under stereomicroscopic guidance. Particular attention

was paid to gently removing the tectorial membrane to ensure complete exposure of the hair cell bundles while preserving their structural integrity.

For SEM preparation, samples were sequentially dehydrated in a graded ethanol series (30 to 100%), critical-point dried with liquid CO_2, and sputter-coated with a 5-nm-thick platinum layer. The coating thickness was monitored in real-time with a quartz crystal microbalance. Specimens were imaged with a field-emission SEM (FEI Quanta 250) operated at parameters optimized for high-resolution visualization of cochlear ultrastructure, with a principal focus on the morphological details of hair cell bundles.

Transmission electron microscope (TEM). Primary fixation was performed on cochlear tissues from WT and *Kcnh8*$^{-/-}$ mice using 2.5% glutaraldehyde in 0.1 M phosphate buffer (Vokai Biotech) for at least 3 hours. After buffer rinses, the specimens were post-fixed in 1% osmium tetroxide (Sigma-Aldrich), dehydrated in a graded ethanol series, and embedded in epoxy resin. The resulting blocks were sectioned into 50 nm ultrathin slices, which were collected on copper grids and stained sequentially with 4% uranyl acetate (Sigma-Aldrich) and 0.4% (macklin) lead citrate. A Philips CM120 transmission electron microscope was used to examine the ultrastructure, with a specific focus on IHC ribbon synapses and vesicle populations.

Electrophysiological recordings. To evaluate the electrophysiological properties of native IHCs in WT, *Kcnh8* knockout (*Kcnh8*-KO), and *Kcnh8*-overexpressing (*Kcnh8*-OE) mice, whole-cell patch-clamp recordings were performed on acutely isolated organs of Corti. The cochlear epithelium containing the apical and middle turns was dissected and transferred to an oxygenated extracellular solution containing (in mM): 125 NaCl, 5.8 KCl, 1.3 $CaCl_2$, 0.9 $MgCl_2$, 0.7 $NaH_2PO_4 \cdot H_2O$, 10 HEPES, 5.6 D-glucose, and 2 Na-pyruvate (290 mOsm, pH 7.40). Hair cells at the apical–middle turn

junction were subjected to patch-clamp recordings. IHCs were visualized under a 60× water-immersion objective on an upright microscope (Olympus), and recordings were made using an EPC10/2 amplifier controlled by Patchmaster (HEKA Electronics). Recording pipettes (borosilicate glass, Sutter) were filled with an internal K^+-based solution (in mM): 110 K-methanesulfonate, 20 KCl, 2 EGTA, 10 Na-phosphocreatine, 10 HEPES, 3 Mg-ATP, and 0.5 Na-GTP (290 mOsm, pH 7.30). Total outward K^+ currents were evoked by stepping from –80 mV to test potentials between –90 mV and +40 mV, followed by a repolarizing step to –110 mV. To emphasize Kv12.1-mediated currents, 5 mM 4-aminopyridine (4-AP) was included in the extracellular solution. For current-clamp experiments, cells were held near –80 mV by injecting a small negative holding current. Resting membrane potential and spontaneous activity were assessed at I = 0, and evoked firing was elicited by 250-ms current steps from –30 to +100 pA. To record Ca^{2+} currents (I_{Ca}) and vesicle exocytosis, the bath solution was switched to a high-Ca^{2+} medium containing (in mM): 125 NaCl, 2.8 KCl, 5 $CaCl_2$, 1 $MgCl_2$, 10 HEPES, 5.6 D-glucose, and 2 Na-pyruvate (290 mOsm, pH 7.40). Pipettes were filled with a Cs^+-based internal solution (in mM): 135 Cs-methanesulfonate, 10 CsCl, 10 TEA-Cl, 2 EGTA, 10 HEPES, 3 Mg-ATP, and 0.5 Na-GTP (290 mOsm, pH 7.20). Pipettes were coated with dental wax to minimize stray capacitance. I_{Ca} was obtained using a voltage-ramp protocol from –90 mV to +70 mV over 300 ms. Exocytosis was quantified using the Patchmaster "Sine+DC" lock-in method. A 1-kHz, 50-mV (peak-to-peak) sinusoid was superimposed on the holding potential before and after 200-ms depolarizing pulses to 0 mV. Whole-cell capacitance (Cm) was extracted from the resulting admittance signal, and the stimulus-induced change (ΔCm) was used as a measure of vesicle fusion. All recordings were conducted at room temperature. Liquid junction potentials (~10 mV) were calculated and corrected offline.

Electrophysiological analysis. Patch-clamp recordings were acquired using HEKA hardware (HEKA) and analyzed with Igor Pro software (Wavemetrics). The voltage dependence of channel activation was determined from steady-state current amplitudes using standard voltage-step protocols. G/G_{max}-voltage (I-V) relationships were depicted by plotting the maximal steady-state current amplitudes against test potentials. These data were fitted with a two-state Boltzmann function: $G = G_{min} + (G_{max} - G_{min}) / (1 + \exp((V - V_h)/s))$, where G is the normalized conductance, V is the test potential, V_h represents the voltage for half-maximal activation, and s means the slope factor. Steady-state Potassium current (I_k), Calcium currents (I_{Ca}), as well as exocytic capacitance changes (ΔCm) were quantified using a custom Igor Pro program developed by Professor Geng-lin Li's laboratory at Fudan University.

Supplementary Figures

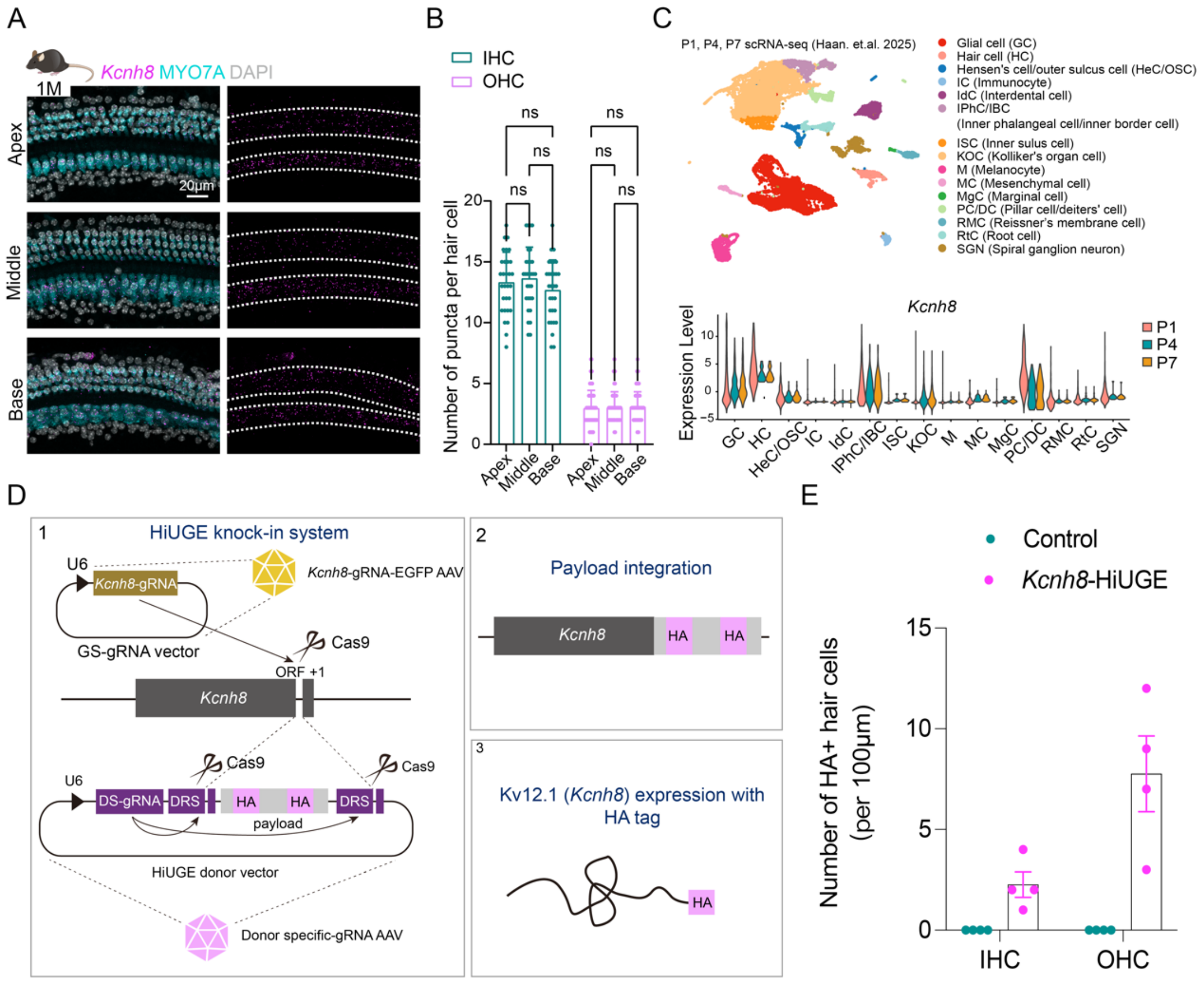

Supplemental Figure 1. *Kcnh8* expression in the mouse cochlea and design of a *Kcnh8*-HA knock-in strategy. (**A**) Representative confocal images showing *Kcnh8* mRNA expression in the apex, middle, and basal turns of the whole-mount mouse cochlea tissues at 1M. HCs are stained with MYO7A (grey). Nuclei are stained with DAPI (grey). Scale bars, 20 μm. (**B**) Quantification of *Kcnh8* RNA puncta per IHC (green) and OHC (magenta) from the staining shown in (**A**). 30 IHCs and 100 OHCs from 3 mice per groups. (**C**) The violin plot shows the *Kcnh8* expression pattern in the P1–P7 cochlear scRNA-seq data. The upper UMAP displays the distribution of each cluster. (**D**) Schematic of the HiUGE knock-in method used to tag endogenous *Kcnh8* with an HA epitope. (**E**) Quantification of HA-positive hair cells after *Kcnh8*-HiUGE transfection.

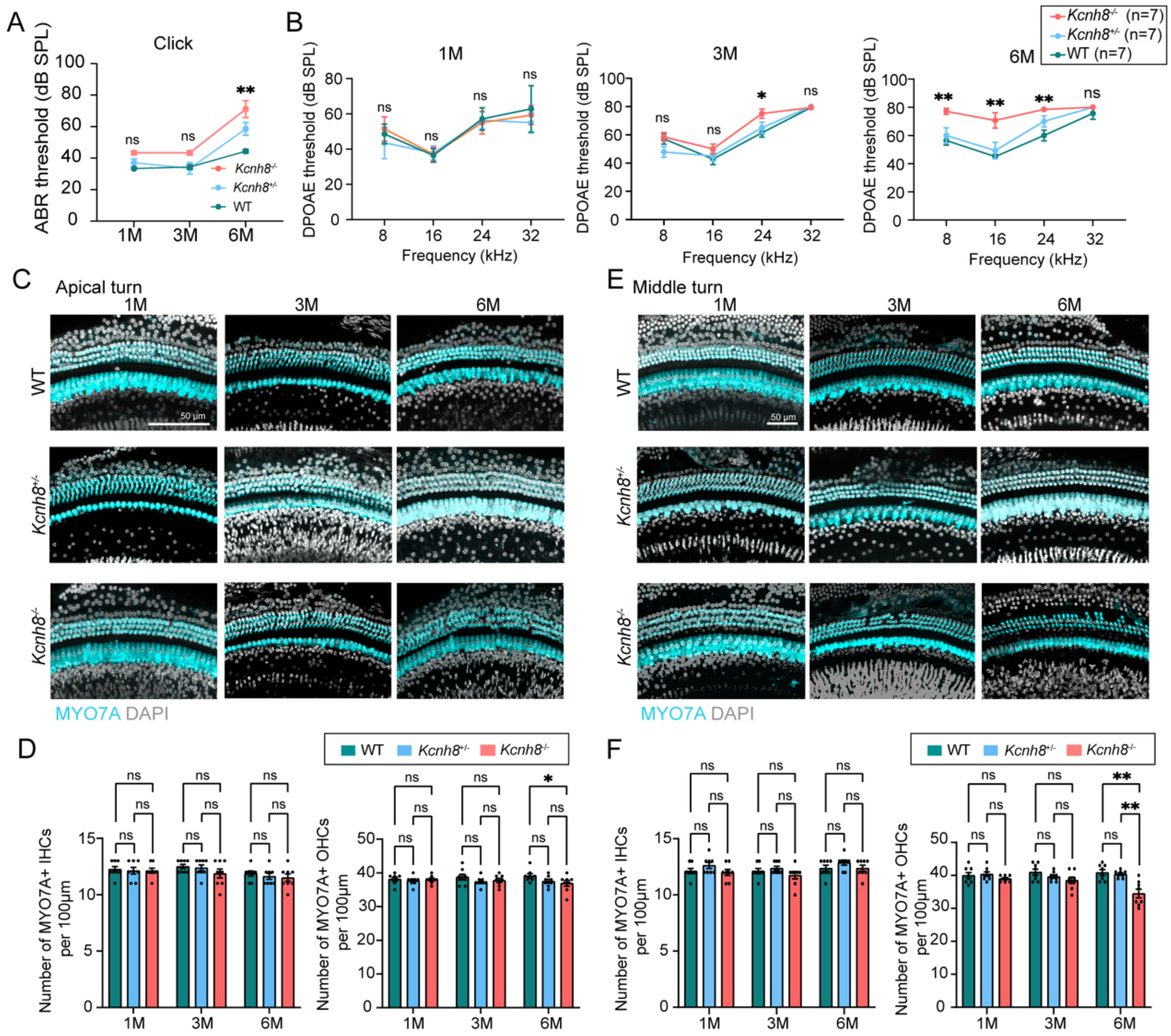

Supplemental Figure 2. ***Kcnh8*** **deficiency causes OHC loss and hearing dysfunction.** (**A**) ABR thresholds in response to click stimuli in WT, *Kcnh8*$^{+/-}$, and *Kcnh8*$^{-/-}$ mice at 1, 3, and 6 months. (**B**) DPOAE threshold in WT, *Kcnh8*$^{+/-}$, and *Kcnh8*$^{-/-}$ mice at 1 month, 3M, and 6 M. The number (n) of mice was indicated in each group. (**C**) Representative confocal images of HCs in the apex turn of WT, *Kcnh8*$^{+/-}$, and *Kcnh8*$^{-/-}$ mice cochlea at 1M, 3M and 6M. The tissue was co-stained with MYO7A (cyan) and DAPI (grey). (**D**) Quantification of MYO7A+ IHCs (left panel) and MYO7A+ OHCs (right panel) from images in (**C**) (n= 8 mice for each group). (**E**) Representative confocal images of HCs in the middle turn of WT, *Kcnh8*$^{+/-}$, and *Kcnh8*$^{-/-}$ mice cochlea at 1M, 3M and 6M. (**F**) Quantification of MYO7A+ IHCs (left panel) and MYO7A+ OHCs (right panel) from images in (E). Data are presented as means ±

SEMs. Statistical significance was determined using two-way ANOVA with Tukey's post hoc test (*$P < 0.05$, **$P < 0.01$, n.s., not significant). Scale bars are given in (**C**) and (**E**).

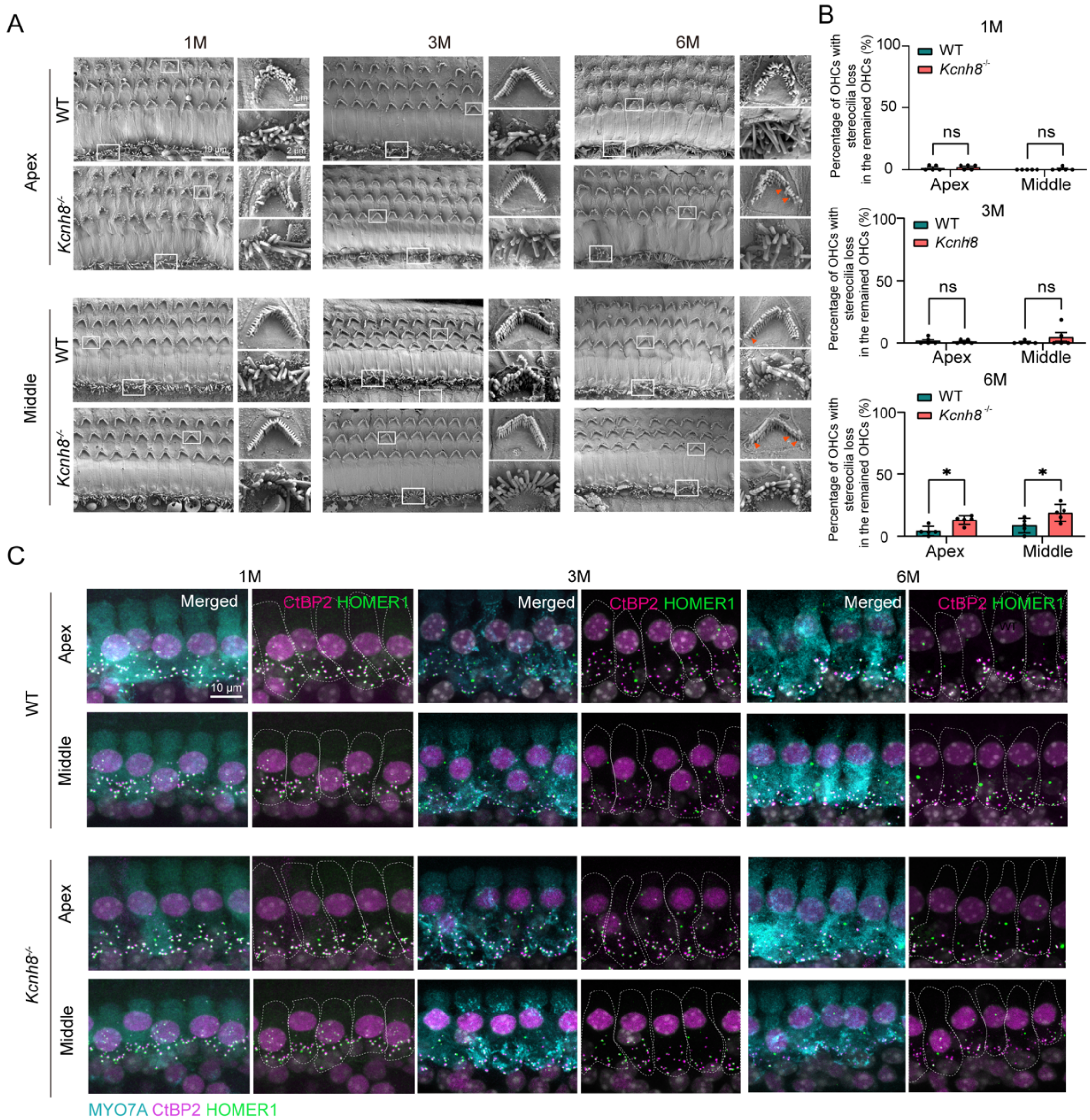

Supplemental Figure 3. ***Kcnh8*** **deficiency leads to progressive HC stereocilia loss and ribbon synapses. (A)** SEM images of the apex and the middle turns of the cochlea and magnified views of stereocilia bundles on IHCs and OHCs from WT and *Kcnh8*$^{-/-}$ mice at 1, 3, and 6 months. **(B)** Quantification of the percentage of stereocilia loss in OHCs from the images in (**A**) (n=5 mice per group). **(C)** Representative confocal images of IHC ribbon synapses from the apex and middle turn in WT and *Kcnh8*$^{-/-}$ mice at 1, 3, and 6 months. Tissues were co-stained for MYO7A (cyan), CtBP2 (magenta), and HOMER1 (green). Statistical significance was determined using two-way ANOVA

with Tukey's post hoc test, as appropriate (*$P < 0.05$, n.s., not significant). Scale bars are provided in (**A**) and (**C**).

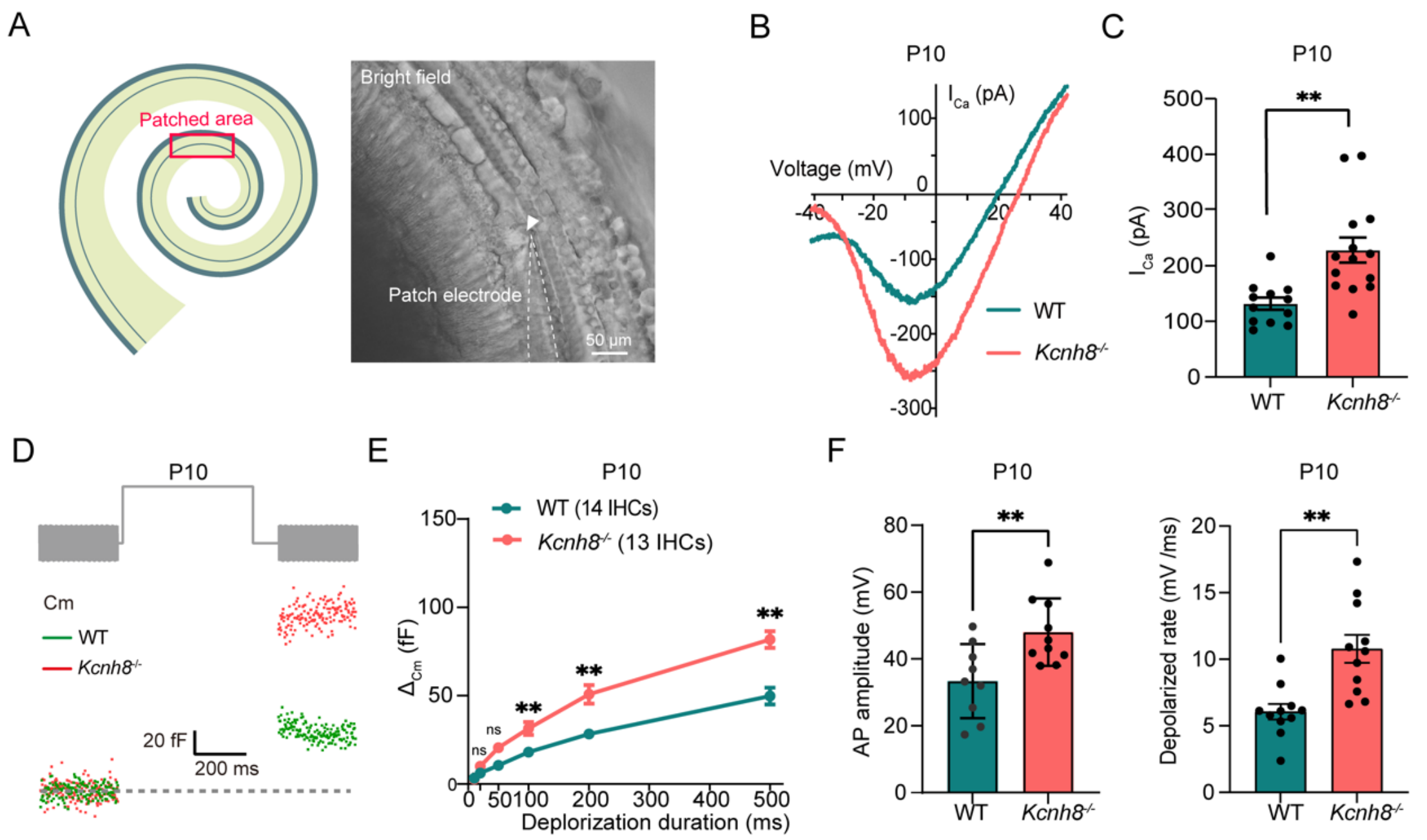

Supplemental Figure 4. ***Kcnh8***$^{-/-}$ **HCs exhibit hyperexcitability and enhanced synaptic transmission at P10.** (**A**) The schematic shows the patch location at the apical-middle turn junction, with a representative IR-DIC image of an IHC patch-clamp recording. (**B**) Representative voltage-clamp recordings of evoked Ca^{2+} currents in IHCs from P10 WT and *Kcnh*$^{-/-}$ mice. (**C**) Quantification of peak evoked Ca^{2+} current in IHCs from P10 WT and *Kcnh8*$^{-/-}$ IHCs from recordings in (B). (D) Representative recordings of ΔCm in response to a 500 ms depolarizing pulse in IHCs from P10 WT and *Kcnh8*$^{-/-}$ mice. (E) Summary of ΔCm evoked by depolarizing pulses of varying durations (10–500 ms) in P10 WT and *Kcnh8*$^{-/-}$ IHCs from 5 mice. (F) Quantification of action potential (AP) peak amplitude (left panel) and depolarization rate (right panel) in IHCs from P10 WT and *Kcnh8*$^{-/-}$ IHCs. Each dot represents one IHC from 5 mice per group (**C**) and (**F**). Statistical significance was determined using unpaired t-test, as appropriate (**$P < 0.01$, n.s., not significant). Scale bars are provided in (**A**).

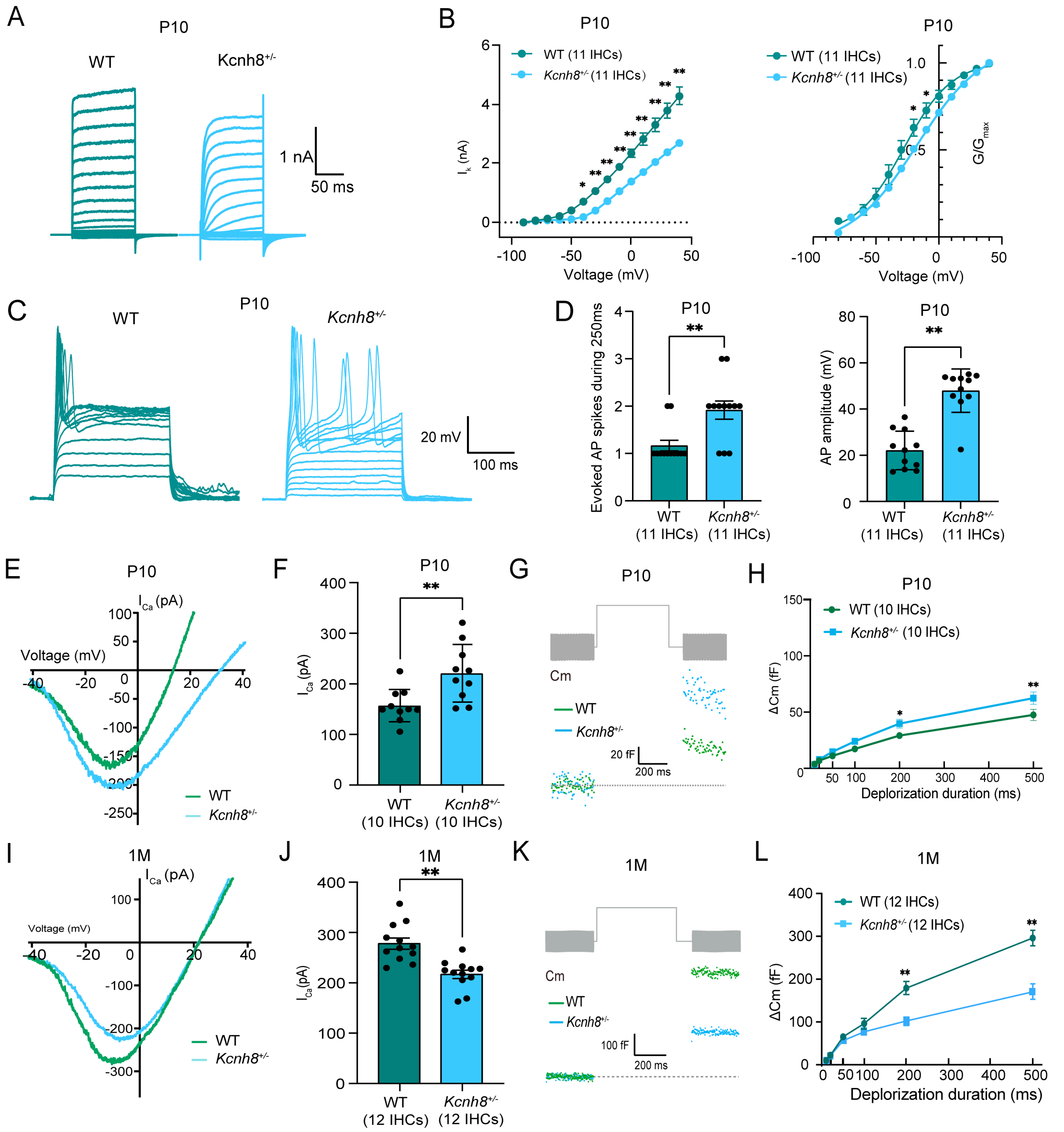

Supplemental Figure 5. ***Kcnh8*$^{+/-}$ HCs exhibit hyperexcitability at P10 and decreased synaptic vesicle release function in adulthood.** (**A**) Representative whole-cell K^+ current traces recorded from P10 WT and *Kcnh8*$^{+/-}$ IHCs. (**B**) Steady-state K^+ currents and voltage dependence of activation from P10 WT and *Kcnh8*$^{+/-}$ IHCs from 5 mice. (**C**) Representative current-evoked action potentials recorded from P10 WT and *Kcnh8*$^{+/-}$ IHCs. (**D**) Quantification of the number of evoked APs (left) and peak amplitude of the first evoked AP (right) from P10 WT and *Kcnh8*$^{+/-}$IHCs from 5 mice with 80 pA current injection during 250 ms. (**E**) Representative I–V curves of Ca^{2+} currents recorded from P10 WT and *Kcnh8*$^{+/-}$ IHCs. (**F**) Quantification of the peak Ca^{2+}

current from P10 WT and *Kcnh8*$^{+/-}$ IHCs from 6 mice. (**G**) Analysis of changes in membrane capacitance (ΔCm) evoked by depolarizing pulses (10–500 ms) in P10 WT and *Kcnh8*$^{+/-}$ IHCs from 6 mice. (**H**) Representative ΔCm evoked by a 500-ms depolarizing pulse in P10 WT and *Kcnh8*$^{+/-}$ IHCs. (**I**) Representative I–V curves of Ca^{2+} currents recorded from 1M WT and *Kcnh8*$^{+/-}$ IHCs. (**J**) Quantification of the peak Ca^{2+} current from 1M WT (n=12) and *Kcnh8*$^{+/-}$ IHCs from 6 mice. (**K**) Analysis of ΔCm evoked by depolarizing pulses (10–500 ms) in 1M WT and *Kcnh8*$^{+/-}$ IHCs from 6 mice (**L**)Representative ΔC_m evoked by 500-ms depolarized pulse in 1M WT and *Kcnh8*$^{+/-}$ IHCs. Data are reported as mean ± SEM. Statistical significance was determined by unpaired *t*-test or two-way ANOVA with Tukey's *post hoc* test (*$P < 0.05$, **$P < 0.01$; n.s., not significant). The number of patched IHCs was indicated in each group.

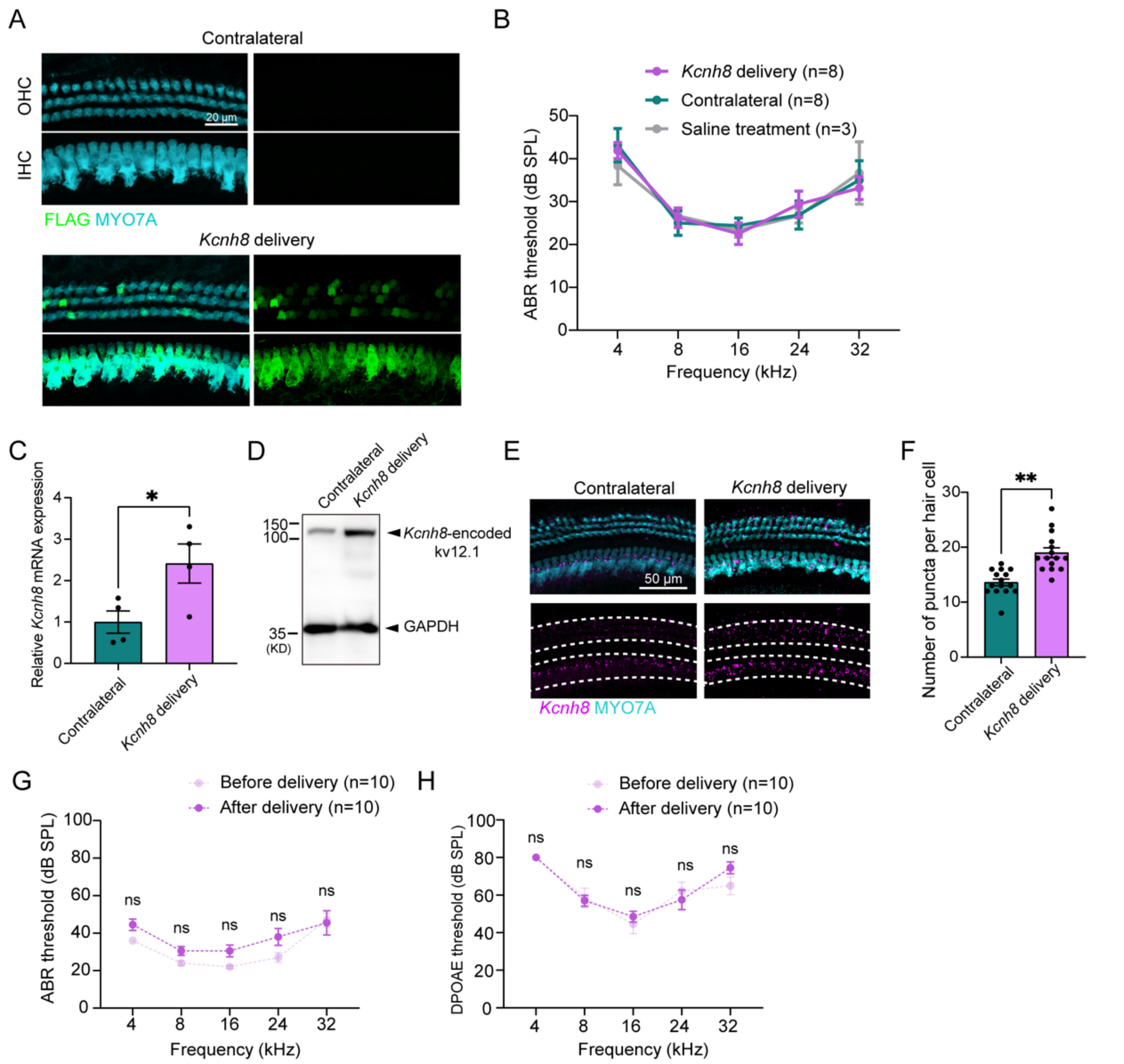

Supplemental Figure 6. Efficient and specific transduction of *Kcnh8* into HCs mediated by AAVanc80L65 in vivo. (**A**) Representative confocal images of HCs from the middle turn of the *Kcnh8*-delivery ear and the contralateral ear of 1M mice. Tissues were co-stained for FLAG (green) to detect the *Kcnh8*-encoded protein and MYO7A (cyan) to label HCs. Scale bars, 20 µm. (**B**) ABR thresholds from ears treated with *Kcnh8*-delivery, their contralateral ears, and ears from saline-treatment mice. The number (n) of mice was indicated in each group. (**C**) RT-PCR showing *Kcnh8* expression in delivery vs contralateral ears of 1M mice. (**D**) WB analysis of Kv12.1 protein in *Kcnh8*-delivered vs contralateral ears. (**E**). Representative images of *in situ* validation of *Kcnh8* expression in *Kcnh8*-delivery ear and the contralateral ear of 1M

mice. Scale bars, 50 μm. (**F**). Qualification of *Kcnh8* mRNA puncta per IHC from the images in (**E**). 15 IHCs were harvested from 3 mice. (**G**). ABR thresholds before (light magenta) and after (dark magenta) *Kcnh8* delivery in *Kcnh8*-delivery ear of 1M mice (n= 10). (**H**). DPOAE thresholds before (light magenta) and after (dark magenta) *Kcnh8* delivery in *Kcnh8*-delivery ear of 1M mice (n= 10). Data are reported as mean ± SEM. Statistical significance was determined by unpaired *t*-test or two-way ANOVA with Tukey's *post hoc* test (**P* < 0.05, ***P* < 0.01; n.s., not significant).

Supplemental Tables

Table. S1. PCR Primers

Primers for genotyping		
**Gene**	**Forward Primer**	**Reverse primer**
Kcnh8 WT	5'-CATGGACACATGGTTGGGAAGTTCT-3'	5'-CACCTGAAGGAAGGCTTGTTTCATC-3'
Kcnh8 Mut	5'-CTCCAGACAGAACCAAAGGAAGATC-3'	5'- ATGCCTCGTACTGCTGAAGTTTCC-3'
**Primers for RT-PCR**		
**Gene**	**Forward Primer**	**Reverse primer**
Kcnh8	5'-CATCTTGTCACCTCAGCAGCCATC-3'	5'-AGCACTCCAACTCACCCTAGTCTG-3'
Gapdh	5'-CATCACTGCCACCCAGAAGACTG-3'	5'-ATGCCAGTGAGCTTCCCGTTCAG-3'

Table. S2. RNAscope probes

Probes	Vendor	Identifier
RNAscope™ Probe- Mm-*Kcnh8*	Advanced Cell Diagnostics	Cat#: 546641
RNAscope™ Probe- Hs-*Kcnh8*-C1	Advanced Cell Diagnostics	Cat#: 1188971-C1

Table. S3. Assay kits

kits	Vendor	Identifier
RNAscope Multiplex Fluorescent Reagent Kit v2	Advanced Cell Diagnostics	Cat#: 323100
PrimeScript™ RT-PCR Kit	TaKaRa	Cat#: RR014A

Table. S4. Antibodies and reagents

**Antibodies**	**Vendor**	**Identifier**
anti-Kv12.1 (1:1000)	Thermo Fisher Scientific	Cat#: PA5-68690;<br>RRID: AB_2691291
anti-MYO7A (1:400)	Proteus BioSciences	Cat#: 25-6790;<br>RRID: AB_10015251
anti-CtBP2 (1:400)	BD Biosciences	Cat#: 612044<br>RRID: AB_399431
anti-Homer1 (1:100)	BD Biosciences	Cat#: sc-55463<br>RRID: AB_629655
anti-Homer1 (1:400)	Synaptic Systems	Cat# 160 006;<br>RRID: AB_2631222
anti-SOX2 (1:400)	BD Biosciences	Cat#: 560291<br>RRID: AB_1645334
anti-FLAG (1:200)	Proteintech	Cat#: 66008-4-Ig<br>RRID: AB_2918475
GAPDH-Loading Control mouse Monoclonal Antibody HRP Conjugated (1:20000)	Signalway Antibody	Cat#: C92848HRP<br>RRID: AB_2537659
Alexa 555-conjugated anti-rabbit secondary antibody (1:500)	Thermo Fisher Scientific	Cat#: A-31572;<br>RRID: AB_162543
Alexa 647-conjugated anti-chicken secondary antibody (1:500)	Thermo Fisher Scientific	Cat#: A-21447;<br>RRID: AB_2535866

Alexa 488-conjugated anti-mouse secondary antibody (1:500)	Thermo Fisher Scientific	Cat# A-11059; RRID: AB_142495
HRP-conjugated anti-rabbit secondary antibody (1:2000)	Thermo Fisher Scientific	Cat#: PA1-85597; RRID: AB_933869
**reagents**	**Vendor**	**Identifier**
DMSO	Sigma-Aldrich	Cat#: D2438
EPG-2AM	Maokang biotec	Cat#: MX4513-50UG
EDTA	SIMUWU biotech	Cat#: SD0279-500G
PFA	Sigma-Aldrich	Cat#: 158127-500G
Agarose LE	TIANGEN biotech	Cat#: B0627A01
Sucrose	SIMUWU biotech	Cat#: SD0259-500G
Triton X-100	Sigma-Aldrich	Cat#: X100
DAPI	Invitrogen	Cat#: D1306
MitoSO™ Red	Invitrogen	Cat#: M36005
CM-H_2DCFDA	Beyotime biotech	Cat#: S0035S-1
FM 1-43FX	Invitrogen	Cat#: F35355
Goat serum	Sangon biotech	Cat#: E510009-0100
Zoletil 50	Virbac	Cat#: BN 9GRRA
dexmedetomidine hydrochloride	DEXDOMITOR	Cat#: 138445-4

Table. S5. Software and algorithms

Software& Programming	Vendor	Sources
SigGenRZ	Tucker-Davis Technologies	https://www.tdt.com/product/biosigrz-abr-dpoae-software/
Fiji	National Institutes of Health	https://imagej.net/software/fiji/#publication
PatchMaster	HEKA Elektronik	https://www.heka.com/
Igor	Wavemetrics	https://www.wavemetrics.com/products/igorpro
GraphPad Prism (v.6.0)	GraphPad	https://www.graphpad.com/
Igor Programs	Geng-lin Li's Lab	NA
