# Supplementary material for: Kv12.1 (Kcnh8) maintains hair cell potassium homeostasis and protects against noise-induced hearing loss in mice
Source: J Clin Invest. 2026 Nov 16;136(22):e202988. doi: 10.1172/JCI202988 (PMC13767643; doi:10.1172/JCI202988)
Supplement: Supplementary file 2 [file jci-136-202988-s240.pdf]

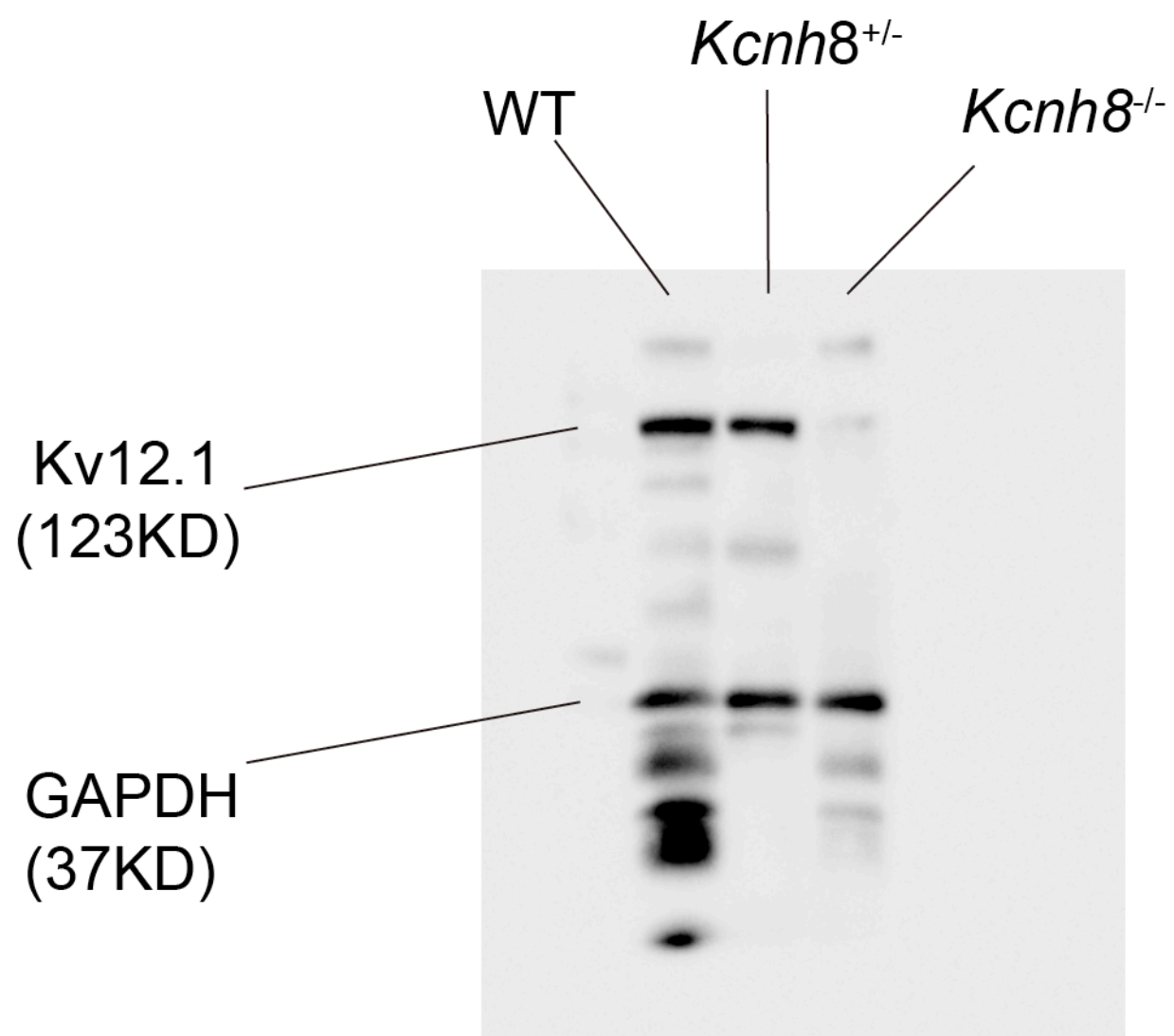

Full unedited gel for Figure 2C

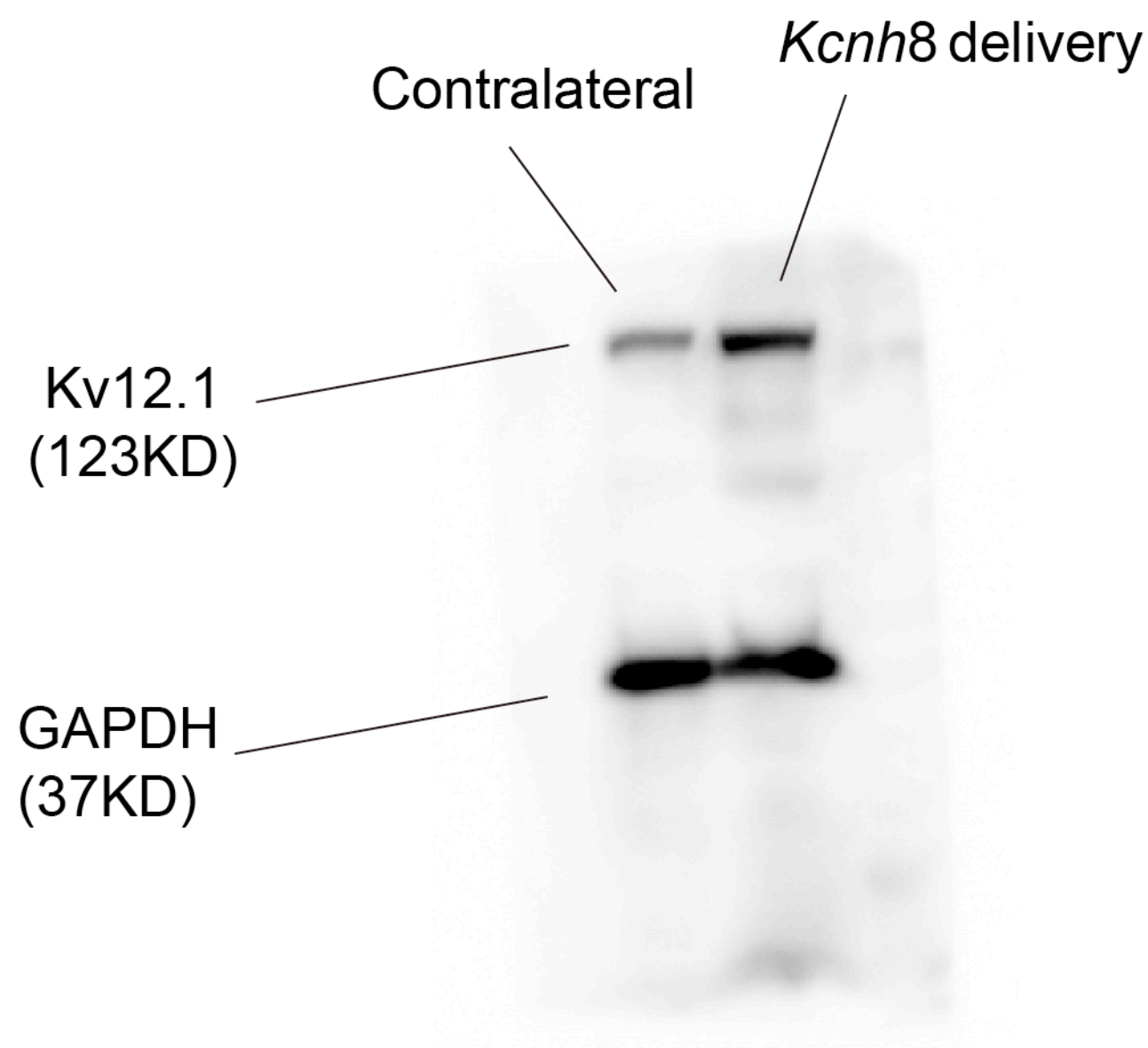

Full unedited gel for Figure S6D
